# Highly diverse ribonucleic acid viruses in the viromes of eukaryotic host species in Yunnan province, China

**DOI:** 10.3389/fmicb.2022.1019444

**Published:** 2022-10-13

**Authors:** Zhenzhi Han, Jinbo Xiao, Yang Song, Xiaonan Zhao, Qiang Sun, Huanhuan Lu, Keyi Zhang, Jichen Li, Junhan Li, Fenfen Si, Guoyan Zhang, Hehe Zhao, Senquan Jia, Jienan Zhou, Dongyan Wang, Shuangli Zhu, Dongmei Yan, Wenbo Xu, Xiaoqing Fu, Yong Zhang

**Affiliations:** ^1^National Laboratory for Poliomyelitis, WHO Western Pacific Region Office (WPRO) Regional Polio Reference Laboratory, National Health Commission (NHC) Key Laboratory for Biosafety, NHC Key Laboratory for Medical Virology, National Institute for Viral Disease Control and Prevention, Chinese Center for Disease Control and Prevention, Beijing, China; ^2^Laboratory of Virology, Beijing Key Laboratory of Etiology of Viral Diseases in Children, Capital Institute of Pediatrics, Beijing, China; ^3^Yunnan Center for Disease Control and Prevention, Kunming, China; ^4^Center for Biosafety Mega-Science, Chinese Academy of Sciences, Wuhan, China

**Keywords:** metagenomic next-generation sequencing (mNGS), virome, RNA viruses, picornavirus, coronavirus, viral evolution

## Abstract

**Background:**

The diversity in currently documented viruses and their morphological characteristics indicates the need for understanding the evolutionary characteristics of viruses. Notably, further studies are needed to obtain a comprehensive landscape of virome, the virome of host species in Yunnan province, China.

**Materials and methods:**

We implemented the metagenomic next-generation sequencing strategy to investigate the viral diversity, which involved in 465 specimens collected from bats, pangolins, monkeys, and other species. The diverse RNA viruses were analyzed, especially focusing on the genome organization, genetic divergence and phylogenetic relationships.

**Results:**

In this study, we investigated the viral composition of eight libraries from bats, pangolins, monkeys, and other species, and found several diverse RNA viruses, including the Alphacoronavirus from bat specimens. By characterizing the genome organization, genetic divergence, and phylogenetic relationships, we identified five Alphacoronavirus strains, which shared phylogenetic association with Bat-CoV-HKU8-related strains. The pestivirus-like virus related to recently identified Dongyang pangolin virus (DYPV) strains from dead pangolin specimens, suggesting that these viruses are evolving. Some genomes showed higher divergence from known species (e.g., calicivirus CS9-Cali-YN-CHN-2020), and many showed evidence of recombination events with unknown or known strains (e.g., mamastroviruses BF2-astro-YN-CHN-2020 and EV-A122 AKM5-YN-CHN-2020). The newly identified viruses showed extensive changes and could be assigned as new species, or even genus (e.g., calicivirus CS9-Cali-YN-CHN-2020 and iflavirus Ifla-YN-CHN-2020). Moreover, we identified several highly divergent RNA viruses and estimated their evolutionary characteristics among different hosts, providing data for further examination of their evolutionary dynamics.

**Conclusion:**

Overall, our study emphasizes the close association between emerging viruses and infectious diseases, and the need for more comprehensive surveys.

## Introduction

It is well-known that humans, like other animals, harbor a rich diversity of microorganisms, such as bacteria; moreover, they also harbor a remarkable diversity of viruses (Geoghegan and Holmes, 2017; Shi et al., 2018a; Liang and Bushman, 2021). Compared to humans, there is much greater viral diversity in wild animals, and they frequently harbor many highly-infectious viruses, such as coronaviruses, influenza viruses, and haemorrhagic fever-associated viruses. Although currently documented viruses present remarkable diversity in their morphological characteristics, genomic forms, transmission pathways, and pathogenesis, unknown viruses remain domination in virosphere in the literatures (Geoghegan and Holmes, 2017; Zhang et al., 2018).

Rapid and accurate identification of the etiology at the initial stage of disease spread, especially in large outbreaks, can provide important information about the pathogen and appropriate countermeasures (Lu et al., 2020a; Wu et al., 2020). However, traditional methods, such as cell culture, morphological observations, serum typing, and polymerase chain reaction (PCR), are inherently biased, and a long time may be wasted characterizing the virome (Reyes et al., 2012; Shi et al., 2016a,2018b). With the development and application of metagenome sequencing for viromes, many zoonotic pathogens and novel viruses have been characterized, greatly expanding the number of viruses documented in the literatures, such as coronaviruses, as well as their host profiles (Shi et al., 2016a,2018a,^2019^; Han et al., 2020b; Zhou et al., 2020).

The untargeted sequencing technology called shotgun sequencing was developed to evaluate the entire genetic material in a sample (Breitbart et al., 2003; Edwards and Rohwer, 2005; Bohannon, 2007), and has significantly advanced the analysis of species composition, structure, and function in many ecological environments. Metagenomic next generation sequencing (mNGS) can be used to identify the whole community of a specimen. This method to study viromes can provide basic data regarding viral distribution as well as an assessment of the potential for ecological risks, such as spill over events (Hu et al., 2020; Zhou and Shi, 2021). mNGS has facilitated the identification of novel viruses, especially novel RNA viruses, significantly improving our ability to identify viral pathogens and revealing the unprecedented diversity of RNA viruses, their deep evolutionary scale, and their association with viral diseases (Shi et al., 2018b; Zhang et al., 2018). In humans, the gut virome predominantly consists of bacteriophages, including *Caudovirales* (double-stranded DNA viruses) and *Microviridae* (single-stranded DNA viruses) (Shkoporov and Hill, 2019; Liang and Bushman, 2021). Although the differences in the viromes of several infant cohorts have been reported, the dynamics of host-virome interaction and the related mechanisms await further investigation (Lim et al., 2015; Norman et al., 2015; Moreno-Gallego et al., 2019). Wild animals, which typically harbor more diverse viromes, play a significant role in the spread and evolution of viral diseases, as illustrated by the numerous viral spills over events and outbreaks associated with wild animals (Gao, 2018; Hu et al., 2020; Zhou and Shi, 2021). The more virome projects that are conducted to explore the viromes of wild animals and environments, the better we can assess the ecological risks for potential future disease pandemics.

In this study, various specimens of bats, pangolins, monkeys, and other species, including throat swabs, anal swabs, sera, and tissues, were collected in Yunnan Province, China in February 2020 for mNGS analysis. We identified several novel RNA viruses from NGS libraries generated using these specimens, including alphacoronaviruses from bat specimens. Some of the identified viruses have not been previously analyzed, and some showed higher genetic divergence compared to known virus species. We characterized the genomic organization, genetic divergence, and phylogenetic relationships of these new viruses. Some of the partial novel viruses discovered in this study could be assigned as new species, or even new genera. This study provides valuable basic data for many RNA viruses of different families including the evolutionary characteristics of several families.

## Materials and methods

### Sample collection

A total of 465 specimens, involving various host species such as bats, pangolins, and monkeys, were collected from February 19, 2020 to February 28, 2020 in seven cities and counties in Yunnan province, China (Supplementary Table 1). These specimens were collected from zoos and wild livestock, including numerous species. Most specimens collected included throat swabs, anal swabs, and tissue samples (lung, heart, spleen, muscle, and intestine), as well as a small number of serum samples from breeders or from people in close contact with these animals. Swab samples were collected and stored in RNAlater (Invitrogen, Waltham, MA, USA), while human serum samples were collected and stored in serum collection tubes. Tissues samples were obtained at a local laboratory and stored at −80°C before being sent to our laboratory. Wild animals were sampled for live swabs and subsequently released. All samples were transported on ice and then kept at −80°C.

### Sample processing and metagenomic next-generation sequencing library preparation

The swab specimens were directly subjected to nucleic acid extraction using the QIAamp Viral RNA Mini Kit (Qiagen, Hilden, Germany). For serum and tissue samples, we extracted nucleic acids using Nucleo Spin RNA Blood (MN, Duren, Germany) and RNeasy Plus Mini Kit (Qiagen, Valencia, CA, USA), respectively, following the manufacturer’s instructions. We merged the extracted samples for library preparation, based on host morphological criteria and the geographical distribution of the samples. A total of 18 libraries were constructed using the Illumina TruSeq DNA Preparation Protocol. In brief, the cDNA of each library was synthesized with SuperScript III Reverse Transcriptase (ThermoFisher, Waltham, MA, USA) and N6 random primers, followed by second-strand synthesis with DNA Polymerase I, Large (Klenow) Fragment (ThermoFisher). Each viral sequencing library was prepared following the Illumina TruSeq DNA Preparation Protocol and was sequenced on the NovaSeq 6000 platform (Illumina, San Diego, CA, USA), with the 150 bp paired-end strategy. Library preparation and sequencing were carried out by Guangdong Magigene Biotechnology Co., Ltd., (Guangzhou, China). The meta-transcriptomics sequencing data were submitted to the NCBI Sequence Read Archive (SRA) under accession numbers SRP270853, SRP270853, and PRJNA689958.

### Genome assembly and analysis

Low-quality bases (PHREAD *q* < 20), low complexity sequences, and adaptors from the raw reads were filtered using Trimmomatic software (version 0.39). The remaining reads were aligned to the rRNA database to identify and remove the rRNA reads and generate clean data (Langmead and Salzberg, 2012). These clean data were entered into Centrifuge (version 1.0.4) software for metagenomic classification, and each read was taxonomically assigned (Grabherr et al., 2011). The remaining data were assembled *de novo* using Trinity (version 2.8.4) and Megahit (version 1.2.9) software (Li et al., 2015a). The assembled contigs were mapped against the non-redundant protein database (NR) using BLASTX, with an *e*-value threshold of 1 × 10^–5^. The contigs that had hits in the viral domain were extracted and searched against the nucleotide database (NT) using BLASTN with an *e*-value of 1 × 10^–5^. Ultimately, we obtained eight libraries that contained sufficient viral genomes for analysis, and those that may infect eukaryotic organisms were recorded (Supplementary Table 2). Libraries that did not contain eukaryotic viral genome sequences were removed, even though they contained other viral sequences, such as bacteriophages and other bacterial viruses. The eukaryotic viral genomes, which were the focus of this study, were extracted and analyzed. To obtain high-quality genome sequences, we manually assembled the contigs into scaffolds using reference genome sequences from GenBank and Sequencher (version 5.0). We also mapped the clean reads from the libraries based on the reference genome sequences using BWA to optimize the quality of the assembled genomic data. Finally, we obtained nearly full-length genomes or at least the important coding regions of the viral genomes of interest. The whole genome sequences determined in this study have been deposited in GenBank under accession numbers MT649088, MT649091, MT878534, MT878532, and MW450824–MW450843.

### Genome annotation and analysis

We inferred the open reading frames (ORFs) of the nearly full-length viral genomic sequences using ORFfinder software. The ORFs and deduced amino acid sequences of the viruses were obtained, and the ORFs of viruses that contained several segments were also identified. For viral genomes that showed significant divergence compared with known viruses, we tried to locate the major protein domains of the novel viruses using RPS-BLAST against the Conserved Domain Database (CDD) (Lu et al., 2020b). The major conserved domains, including the RNA-dependent RNA polymerase (RdRp), helicase, 3C cysteine protease, and capsid protein domains, were identified. For the viral genomes that showed genomic similarity to known viruses, we collected these neighbors, including the reference genomes from different virus families, and analyzed their phylogenetic relationships. Implementing this strategy, we explored their taxonomical level, phylogenetic associations, and evolutionary dynamics compared with currently circulating viral strains.

For the phylogenetic analyses, we generated genomic sequence alignments using the E-INS-I algorithm in MAFFT (version 7.407) (Katoh and Standley, 2013). For the novel viral genomes that showed low amino acid sequence identity compared with known viruses, the residual regions of ambiguously aligned domains were used as input for TrimAl, to remove the ambiguous regions (Capella-Gutierrez et al., 2009). For viral genomes that showed genomic sequence identity with known viruses, we inferred the phylogenetic dynamics using genomic sequences, including GenBank reference sequences. The optimal nucleotide or amino acid substitution models were inferred using ModelFinder with the Bayesian information criterion (BIC), and then maximum likelihood phylogenetic trees were constructed using IQ-TREE (Nguyen et al., 2015; Zhang et al., 2019). The bootstrap test and SH-like approximate likelihood ratio test (SH-aLRT) were used, with 1,000 replicates for phylogenetic inference. To obtain better diagrams, we manipulated the topology of the maximum likelihood phylogenetic trees using the ggtree package and Figtree (Suchard et al., 2018; Yu et al., 2018). SimPlot (version 3.5.1) was used for recombination analysis, with a 200-nucleotide window moving in 20-nucleotide steps (Salminen et al., 1995). The Recombinant Detection Program (RDP4, v4.46) was used to screen for recombination signals in the dataset using seven methods, RDP, GENECONV, MaxChi, Bootscan, Chimaera, SiScan, and 3Seq (Martin et al., 2015). The statistic significance was adopted when the *p* value < 0.05.

### Viral detection by quantitative real-time polymerase chain reaction

To confirm the results of mNGS analysis, real-time RT-PCR assays were used to detect specific viral genomes in each library. The primers and probes, which were designed based on genome contig sequences obtained from the mNGS, are listed in Supplementary Table 3. The assays were performed using the One Step PrimeScript™ RT-PCR Kit (TaKaRa, Shiga, Japan) according to the manufacturer’s protocol. The amplification conditions were as follows: 30 min at 42°C and 10 min at 95°C, followed by 40 cycles of 15 s at 95°C and 45 s at 50°C. Fluorescence was recorded during the 50°C phase.

### Ethics approval and consent to participate

For human samples, written informed consent for the use of their clinical samples was obtained for health purposes at the time of sample collection, and collection procedures were performed at a local hospital. In brief, when the investigators collected the clinical samples at the hospital, the subjects were explained about the use of their clinical samples and signed a written informed consent allowing the analysis of their clinical samples. The study co-ordinators performed the analysis of clinical samples for public health purposes. For non-human subjects studies, the collection of specimens was approved by the local Centre for Diseases Control and Prevention ethics committee to allow investigation in this study. All necropsy and sample collection procedures were performed in strict accordance with the China CDC guidelines for the Laboratory Animal Use and Care [SYXK(Jing)2017-0021]. The study was also approved by the Animal Ethics Review Committee of the National Institute for Viral Diseases Control and Prevention (IVDC), Chinese Centre for Diseases Control and Prevention. All experimental protocols were approved by the IVDC and methods were carried out according to approved guidelines (Blake et al., 2018).

## Results

### Characteristics of the viromes of the metagenomic next-generation sequencing libraries

**FIGURE 1:**
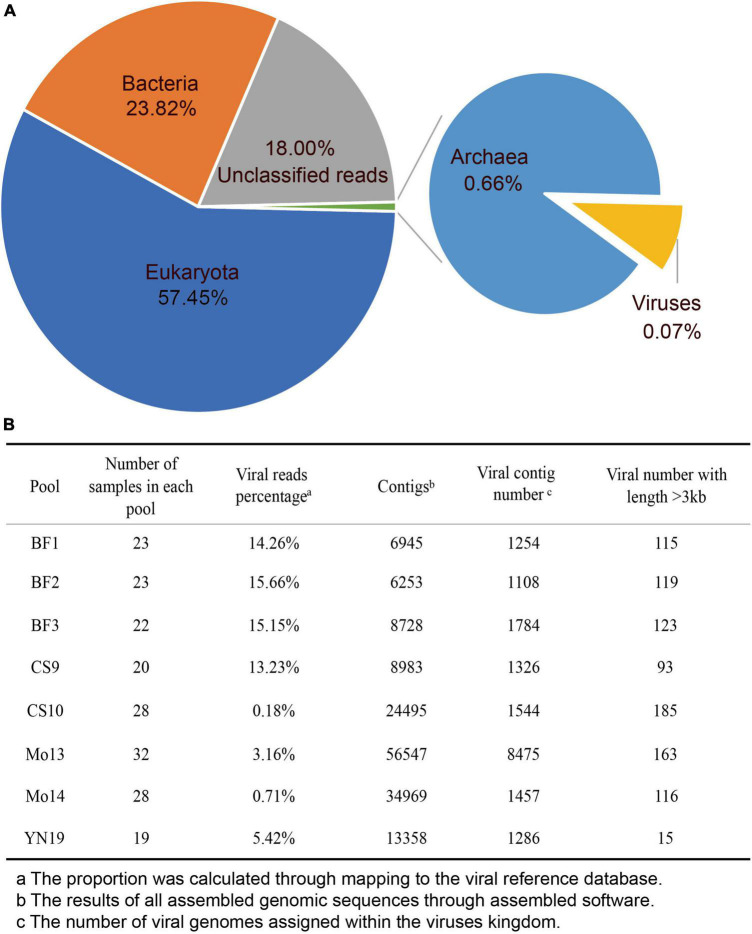
Characteristics of virome data. **(A)** Pie chart of the viral species classifications based on the output data in the eight libraries. **(B)** The assembly statistics and viral taxonomic identifications in each library.

A total of 465 specimens were collected from several species, wherein throat swabs and anal swabs were dominant. All samples were collected in seven cities or counties of Yunnan province, China on Feb, 2020 (Supplementary Table 1). Although we initially attempted to construct 18 libraries during sample processing, some libraries did not include eukaryotic viral genome sequences (e.g., they only contained archaeal and bacterial genomes) or the library could not be established. Ultimately, eight libraries were included in the analysis (Figure 1 and Supplementary Table 2). These eight libraries contained specimens collected from bats, pangolins, monkeys, and other species (Supplementary Table 2). In total, we obtained 1,176,811,635 clean reads from the eight libraries. Of these reads, 57.45% were eukaryotic, 23.82% were bacteria (Figure 1A), and 18.00% were unclassified as they were not assigned to any known taxonomical domain and could be artificial chimera. The viral reads comprised 0.07% of the total clean reads (805,485 reads), representing a rare proportion of the output data.

**FIGURE 2:**
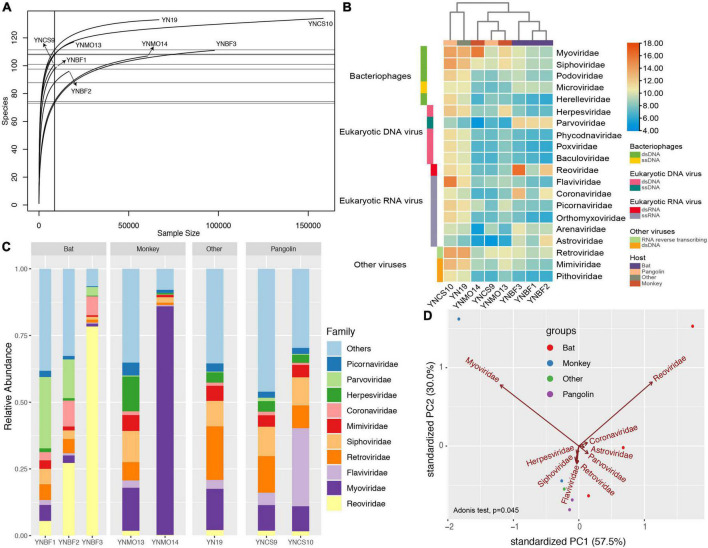
Virome differences among the eight libraries based on read counts. **(A)** Rarefaction curves for each library, with the *x*-axis showing the sample size of each library and *y*-axis showing the viral species identified. **(B)** Heatmap showing the normalized abundance of the eight libraries at the family level. The red columns represent high abundance, and the blue columns represent low abundance. The taxonomical information was list in the right panel. **(C)** Virome composition at the family level. The top 10 virus families are shown. **(D)** Principal component analysis (PCA) showing multivariate variation (host information) and the major contributions of different factors (viral families) to PC1 and PC2. The first two PCA were used, and the groups are shown in different colors.

We explored the taxonomical composition and viral distribution within the different hosts in these eight libraries (Figure 2 and Table 1). The rarefaction curves of four libraries (YN19, YNCS10, YNMO14, and YNBF3) were saturated, and had a larger sample size. In contrast, the numbers of viral species in the other four libraries was nearing a plateau (Figure 2A). Regardless of the host species and the bacterial and unclassified reads, the realms Riboviria and Heunggongvirae dominated the viral data, at 41.2 and 29.5%, respectively. At the family level, *Reoviridae* and *Myoviridae* were predominant, while several other families, such as *Flaviviridae*, *Siphoviridae*, *Mimiviridae*, *Coronaviridae*, *Herpesviridae, Parvoviridae*, and *Picornaviridae*, were also present in relatively lower proportions. Libraries YN19 and YNCS10, in which several viral species were identified, harbored high viral abundance, even though the samples were collected from different host species (Figures 2B,C). Library YNBF3 contained a high abundance of the families *Reoviridae* and *Coronaviridae*, while library YNMO14 harbored a high abundance of the families *Myoviridae* and *Luteoviridae.* More viral reads from the family *Astroviridae* were detected in library YNBF2 (Figures 2B,C). In the principal component analysis (PCA), the families *Reoviridae*, *Myoviridae*, *Coronaviridae*, and *Astroviridae* contributed the most to the variability in the libraries (PC1) (57.5%, Figure 2D), whereas the families *Herpesviridae*, *Siphoviridae*, *Flaviviridae*, and *Retroviridae* were the second most important contributors to the variation (PC2) (30%, Figure 2D). No significant clusters of libraries based on host were observed, indicating no remarkable correlation between viruses and their hosts.

**TABLE 1:** The statistics of viral reads assigned to different families.

Realm	Family^a^	Number of assigned reads^b^	Proportion of viral reads
Riboviria	Reoviridae	155526	19%
	Flaviviridae	74161	9%
	Retroviridae	48013	6%
	Coronaviridae	20432	3%
	Picornaviridae	12653	2%
	Caliciviridae	4051	0.5%
	Secoviridae	1438	0.2%
	Astroviridae	7241	0.9%
	Potyviridae	4615	0.6%
Heunggongvirae	Myoviridae	150052	19%
	Siphoviridae	48240	6%
	Herpesviridae	17879	2%
	Podoviridae	8936	1%
	Herelleviridae	7170	0.9%
	Autographiviridae	4530	0.6%
Varidnaviria	Mimiviridae	21367	3%
	Phycodnaviridae	7198	0.9%
	Poxviridae	6362	0.8%
	Adenoviridae	1989	0.2%
Monodnaviria	Parvoviridae	16736	2%
	Microviridae	8053	1%
	Papillomaviridae	3252	0.4%
	Geminiviridae	2597	0.3%
unclassified viruses	Pandoravirus	27031	3%
	Mollivirus sibericum	4277	0.5%
	Pithoviridae	5657	0.7%

^a^The family or species taxon name.

^b^The number of viral reads assigned to this and child taxa.

After *de novo* assembly, we obtained 6,253–56,547 assembled contigs in each of eight libraries, and we annotated 1,108–8,457 assembled viral contigs in each of the eight libraries (Figure 1B). The proportion of viral reads in each library ranged from 0.18 to 15.66%, which was not consistent with the differences in viral contigs (e.g., library Mo13). The number of viral contigs with a length > 3 kb ranged from 15 to 185, which differed sharply among the libraries. We did not observe any variational tendency or association among the indexes of the assembled data, reflecting the complexity of the mNGS output data.

### Ribonucleic acid viruses infecting the eukaryotic host organisms in this study

In addition to the bacteria, phages, and archaeal viruses in the microbiological dataset, we found several viruses that could potentially infect eukaryotic organisms, and we further analyzed their characteristics (Table 2). Although a large of phages or RNA viruses were revealed in previous researches, the viruses infecting the eukaryotic host organisms were finitely investigated. These viruses, which included both nearly full-length genomes as well as partial genomes, covered five orders, and many differed from their closest relatives in GenBank. Some of them could be classified as new species, which were not previously reported. Most of the genomes were highly abundant in the mNGS data. We confirmed their abundance using a contig-specific quantitative real-time PCR (qRT-PCR) method. Below, we describe some of the individual viruses we detected organized by type.

**TABLE 2:** The potential viruses that infect the eukaryotic organisms detected in each library.

Pool	Contigs number^a^	Genome length^b^	Positive specimen^c^	Classification			Closest relative Genbank accession	Identity^e^	Abundance^f^
				Order	Family	Genus or species^d^			
BF1	11	498–1847	1	Picornavirales	Picornaviridae	Kobuvirus	MF947438	87%	598007
	37	306–1847	5	Stellavirales	Astroviridae	Mamastrovirus	EU847148	82%	186360
	36	312–3135	1	Reovirales	Reoviridae	Rotavirus	KX756624	94%	303932
BF2	13	463–5796	NA	Picornavirales	unclassified Picornavirales	unclassified Picornavirales	MF352427	97%	126672
	75	301–5659	14	Stellavirales	Astroviridae	Mamastrovirus	EU847154	81%	185131
	58	447–3521	3	Reovirales	Reoviridae	Rotavirus	KX756624	94%	305803
	41	308–6336	NA	Nidovirales	Coronaviridae	Alphacoronavirus	KJ473800	94%	35167
BF3	17	305–11047	8	Picornavirales	Iflaviridae	Iflavirus	MN560635	69%	103684
	88	317–2555	17	Stellavirales	Astroviridae	Mamastrovirus	NC043102	76%	184799
	45	316–3529	3	Reovirales	Reoviridae	Rotavirus	KX756624	94%	305384
	16	301–28161	NA	Nidovirales	Coronaviridae	Alphacoronavirus	KJ473799	96%	35846
CS9	11	302–7190	2	Picornavirales	Caliciviridae	Bat calicivirus	KJ641701	66%	143474
	7	305–933	NA	Picornavirales	Dicistroviridae	unclassified Dicistroviridae	MN906003	76%	1072386
	5	308–1181	NA	Picornavirales	Polycipiviridae	Sopolycivirus	KX883910	96%	1008335
CS10	16	588–7054	4	Amarillovirales	Flaviviridae	unclassified Flaviviridae	MK636874	78%	157488
MO13	2	7252–7253	5	Picornavirales	Picornaviridae	EV-A122	KT961654	77%	140780
	8	1071–3776	8	Picornavirales	unclassified Picornavirales	Posa-like viruses	LC123275	88%	103763
MO14	3	6929–7143	4	Picornavirales	Picornaviridae	EV-A122	JX627571	78%	141199
	3	2486–3770	5	Picornavirales	unclassified Picornavirales	Posa-like viruses	LC123275	89%	104022
YN19	1	8299	NA	Picornavirales	Caliciviridae	Vesivirus	MF677852	94%	124372

^a^The number of relative genomic contigs identified in each pool.

^b^The length of contigs that match the indicated virus.

^c^Viral pathogens confirmed by contig-specific qRT-PCR assays in the specimens of each library, with the CT value < 35.

^d^Some exact species information was included within the “Classification” column, even though some novel RNA viruses were not identified at the species level.

^e^The genomic sequences identity through the blastn alignment against the NT database.

^f^The abundance level of transcripts detected was fragments per kilobase transcript length per million fragments mapped (FPKM), and the VP1 segments of rotavirus was used for rotavirus abundance assess.

NA represent the non-test for the specimens or assays using the nested RT-PCR for coronavirus.

### Coronaviruses

**FIGURE 3:**
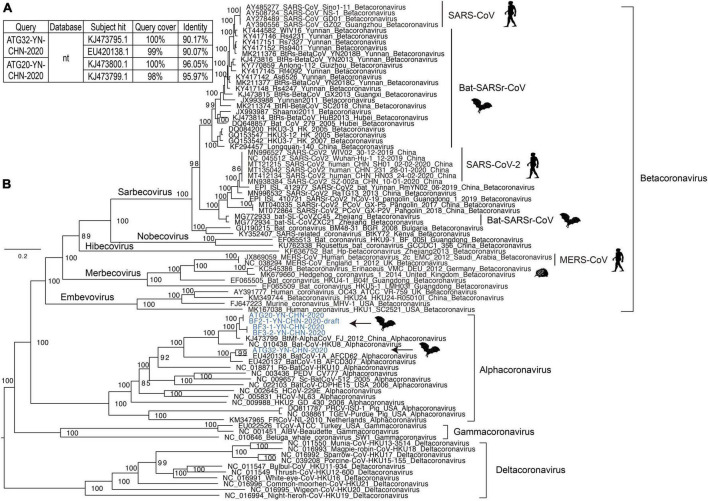
Analysis of coronavirus genomes. **(A)** The top two hits obtained using BLASTN for two representative coronavirus strains. **(B)** Maximum likelihood tree of representative genomes of coronaviruses, including alphacoronaviruses, betacoronaviruses, gammacoronaviruses, and deltacoronaviruses. The new strains identified in this study are shown in blue. The scale bars show the substitutions per site per year. The values at each node indicate the bootstrap and SH-like approximate likelihood ratio test (SH-aLRT) values, with 1,000 bootstrap replicates.

Interestingly, we detected alphacoronavirus contigs in two libraries (BF2 and BF3), and their presence was confirmed by nested PCR with specific primers previously reported to detect these coronaviruses (Poon et al., 2005; Ge et al., 2016). Using this method, we identified five nearly full-length genome sequences of alphacoronaviruses in the specimens, and four were high-quality (Figure 3). All of them clustered with strains of the genus alphacoronavirus in the phylogenetic tree, and they formed two lineages that aggregated with Bat-CoV-HKU8-related coronaviruses (Figure 3B). Although these new viruses clustered with known strains, they actually showed divergence compared with the closest phylogenetic neighbors. This was especially true for strain ATG32-YN-CHN-2020, which showed about 10% genomic divergence when compared with neighboring strains, revealing that these strains are evolving quickly in bat populations (Figure 3A). Four strains in this study, which were detected in different bat specimens, were closely clustered together and shared high identity, suggesting that these strains are co-circulating in local bat populations. The genetic organization and recombination events were also investigated (Supplementary Figure 1), and the major open reading frames (ORFs) in the genomes were identified (e.g., ORF1ab, spike protein, nucleoprotein, membrane protein, and envelope small membrane protein). The analysis showed that their genomic organization was similar to that of other coronaviruses.

Although strain ATG20-YN-CHN-2020 showed high genomic identity with known alphacoronaviruses, it showed relatively lower genomic identity in the partial coding region of the spike protein, and further examination identified a potential recombination event (Supplementary Figure 1, black arrow). Strain ATG32-YN-CHN-2020 showed lower genomic identity with known alphacoronaviruses, although no potential recombination events were detected. Some genomic regions had extremely low identity, such as a segment of ORF1ab and the spike protein coding region. Due to the limited number of alphacoronavirus genomes in the database, a more detailed investigation of the evolutionary dynamics and recombination among these strains was impossible.

### Pestivirus-like viruses

**FIGURE 4:**
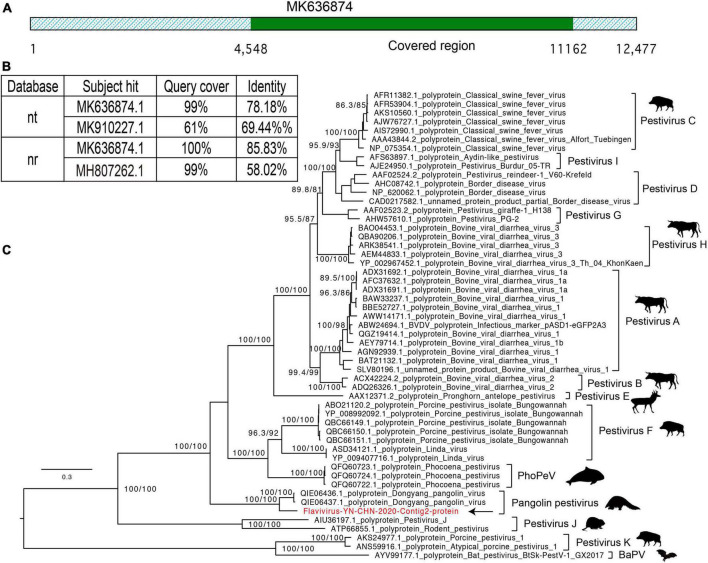
Characterization of the pestivirus genome contigs. **(A)** Graphics of mapped sequences. **(B)** The top two hits in the nucleotide (NT) and protein (NR) databases using the BLASTN and BLASTP algorithms. **(C)** Maximum likelihood tree of the newly identified pestivirus species, neighboring genomes, and newly identified flavivirus, based on the amino acid sequences. The scale bars show the substitutions per site per year, and the values at each node indicate the bootstrap and SH-like approximate likelihood ratio tests (SH-aLRT), with 1,000 bootstrap replicates. The black arrows represent the newly identified strains.

In the CS10 library, we identified 16 contigs that shared nucleotide identity (up to 78%) with Dongyang pangolin virus (DYPV) (GenBank accession no. MK636874.1). The high abundance of pestivirus-like viruses in this library was revealed by its abundant existence in the mNGS output data, and four samples in this library were positive for pestivirus-like viruses (Table 2). Although the CS9 library also contained pangolin specimens, none of them were positive for pestivirus-like viruses by contig-specific qRT-PCR. To obtain the complete genomes of these pestivirus-like viruses, the genetic materials were mapped to the reference genome and manually checked and assembled. However, we were only able to identify a partial genome, 6,584 bp in length (Figure 4A). The genome shared 78.18% genomic identity with DYPV, although some amino acid sequence divergence existed in this genomic region (Figure 4B). Except for two previously reported strains (GenBank accession no. MK636874.1 and MK636875.1), we did not find other strains sharing high genomic identity with strain Flavivirus-YN-CHN-2020 (others had < 60% amino acid identity by BLAST). In the phylogenetic tree, strain Flavivirus-YN-CHN-2020 clustered with two DYPV strains, with a bootstrap value of 100%, and based on the maximum likelihood tree using amino acid sequences, all of they formed a new lineage (Figure 4C). Although they clustered together in the phylogenetic tree, 14.2% amino acid sequence divergence was observed. The results suggest that they may represent a new species of the genus Pestivirus in the family Flaviviridae. Although DYPV is the cause of haemorrhagic diseases in pangolins, which are sometimes fatal (Gao et al., 2020), due to a lack of clinical data, we could not estimate the disease association between this pathogen and any clinical manifestation.

### Picornaviruses

Six families of *Picornavirales* were identified, and they showed high diversity in genomic organization and evolutionary scale (Table 2). Previous studies revealed the abundant genetic diversity, flexible genomic organization, and evolutionary history of these RNA viruses, and showed that picornaviruses were present in higher proportions than other viruses (Shi et al., 2016a,2018a). The specimens analyzed in this study, which were collected from numerous host species, contained several types of picornaviruses (Supplementary Tables 1, 2).

**FIGURE 5:**
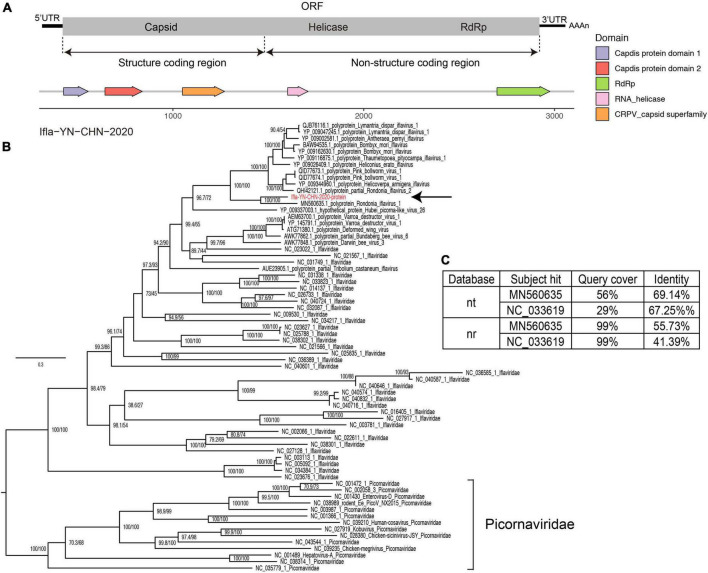
Characterization of iflavirus genome contigs. **(A)** Genomic organization of the detected iflaviruses and annotation of conserved domains. **(B)** Maximum likelihood tree of the complete amino acid sequences of the detected iflaviruses along with reference genomes of known species of *Iflaviridae* and neighboring strains. The scale bars show the substitutions per site per year, and the values at each node indicate the bootstrap and SH-like approximate likelihood ratio tests (SH-aLRT), with 1,000 bootstrap replicates. The black arrows represent the newly identified strains. *Picornaviridae* was used as an outgroup. **(C)** The top two hits in the nucleotide (NT) and protein (NR) databases obtained using BLASTN and BLASTP.

Iflaviruses were identified in library BF3 (Table 2), and we obtained a nearly full-length genome for Ifla-YN-CHN-2020, which shared 69% genomic identity with strain Rondonia_BR_15 (GenBank accession no. MN560635.1). The genome contained an ORF encoding a precursor polyprotein of 3,038 amino acids, which was predicted to be cleaved into several proteins, including capsid protein and non-structural proteins (Figure 5A). An analysis of the genome revealed an organization typical of iflaviruses, which includes the infectious flacherie virus (Valles et al., 2017). The phylogeny showed that Ifla-YN-CHN-2020 was clustered with Rondonia_BR_15, which were detected in samples from bats and ticks, respectively (Figure 5B). The maximum likelihood tree based on the RdRp core confirmed the evolutionary relationships within the family *Iflaviridae* (Supplementary Figure 2). However, analysis of the complete amino acid sequences showed 44.3% divergence (Figure 5C), thus the amino acid divergence between strain Ifla-YN-CHN-2020 and previously reported Iflavirus species is large enough to assign Ifla-YN-CHN-2020 as a new species within the family Iflaviridae, even a novel genus. Due to the lack of demarcation criteria for Iflaviridae from the ICTV, these viruses could be separated into different genera soon (Valles et al., 2017).

Other eukaryotic viruses, such as Mamastroviruses, Rotaviruses, Caliciviruses, and remaining Picornaviruses were described in the Supplementary content, regarding on the limited context (Supplementary content for main text in Supplementary material and Supplementary Figures 3–12).

## Discussion

The development of NGS and associated technologies has enabled the exploration of the composition and functional roles of the species in many ecological environments, in a relatively unbiased way (Quince et al., 2017; Liang and Bushman, 2021). However, the documented virosphere is still dominated by disease-associated viruses, which is a biased view of viral diversity and function (Geoghegan and Holmes, 2017; Zhang et al., 2018). This view might ignore numerous “dormant” viruses that could potentially cause infectious diseases outbreaks in the future. Herein, we investigated the basic genomic characteristics and evolutionary dynamics of a large number of viral species associated with humans and animals, so that we could gather data regarding their ecological niches and estimate their spill over risk. This study reported the virome characteristics of many host species and revealed the evolutionary associations among several novel RNA viruses.

A total of 465 specimens were collected, and the generated libraries contained many novel RNA viruses, which could infect eukaryotic organisms. Although we harvested a large amount of data from each library, the viral reads only accounted for 0.07% of the total reads, and thus represented a rare proportion, which was consistent to previous documents (Ladner et al., 2016; Chang et al., 2020; Han et al., 2020b,2021a; Liang and Bushman, 2021). Although the viromes of the libraries varied dramatically, *Reoviridae*, and *Myoviridae* were the major families in the libraries. The families *Reoviridae*, *Myoviridae*, *Flaviviridae*, *Retroviridae*, *Siphoviridae*, *Mimiviridae*, *Coronaviridae*, *Herpesviridae*, *Parvoviridae*, and *Picornaviridae* were common to all libraries, but their proportions and the numbers of taxonomical assignments differed. *Coronaviridae*, *Astroviridae*, and especially *Reoviridae* and *Myoviridae* contributed most to the variability in PC1, which was consistent with their high proportion of viral reads. *Herpesviridae*, *Siphoviridae*, *Flaviviridae*, and *Retroviridae* were the second most important contributors to the variability in PC2 (30%, Figure 2D). These factors explained most of the variability, up to 87.5%, and illustrated the dominant viral species. The non-significant cluster of libraries based on host group revealed a non-specific correlation between virus and host.

The number of assembled viral contigs also varied among the libraries, and we did not identify any variational tendency among the indexes of assembled contigs and output reads. For example, library Mo13 contained a low proportion of viral reads but greater numbers of assembled contigs. This indicates that a single index could not comprehensively reveal relationship between the mNGS data and the assembled results. The number of sequencing reads, integrity of the assembled genomes, species proportions, and genetic complexity might all significantly influence the statistic index (Li et al., 2015b; Quince et al., 2017). Overall, mNGS yielded more viral species, and we could obtain assembled viral genomes of longer length, compared to Sanger sequencing.

A large number of novel RNA viruses have been characterized, which has expanded our understanding of the evolutionary characteristics of the virosphere, provided models of genomic organization, and revealed their relationships with viral diseases (Shi et al., 2016a,2018a; Gregory et al., 2019; Moreno-Gallego et al., 2019). Interestingly, in this study, we obtained some nearly full-length viral genomes and several fragmented genomes, covering five viral taxonomical orders. To avoid false positive results in this study, we detected the specific viral nucleotides in each specimen using contig-specific qRT-PCR, which confirmed the existence in the raw specimens. For clearly displaying the characteristics of different viral species, we presented the results organized by viral type.

Co-circulation of alphacoronaviruses in the local bat colony was observed, which suggested the possibility of coronavirus recombination within bat populations. Actually, potential recombination events were identified in strain ATG20-YN-CHN-2020; however, we did not identify the recombination donor among the closest neighbors of ATG32-YN-CHN-2020. The evolution and recombination events of coronavirus were reported around the world, which enabled the opportunity for spill over (Hu et al., 2020; Zhou and Shi, 2021). We confirmed the persist evolution of alphacoronaviruses in this study. Because there are relatively few alphacoronavirus genomes in the public database, analyzing the detailed evolutionary dynamics and recombination events proved difficult. More comprehensive, shared, cooperative surveillance of coronavirus is needed to dynamically monitor their evolution and transmission, and not just in human and bat hosts.

Species of astrovirus have been recognized as important pathogens that cause infantile gastroenteritis in humans, dogs, pigs, bats, and other animals (Cortez et al., 2017; Johnson et al., 2017). Our phylogenetic analysis revealed that strain BF2-astro-YN-CHN-2020 was related to other mamastroviruses detected in bat samples. However, in the newly identified strain, ORF2, which encodes the capsid protein in mamastrovirus, showed divergence when compared with other neighboring strains of bat astrovirus. Genomic variations in astrovirus ORF2, which determines the antigenic epitopes of astrovirus particles, have accumulated and would favor host adaption. A recombination event was identified, with an unknown recombination donor, which facilitated the evolution of bat astroviruses. Rotaviruses, which are frequently associated with gastroenteritis in humans and several other animals (Marton et al., 2015; Banyai et al., 2017), were also identified in this study. Strain Rota-BF-YN-CHN-2020 was classified as species rotavirus J in bat, which has been firstly reported in China till now. Compared to another known rotavirus J strains, Rota-BF-YN-CHN-2020 has acquired a nucleotide substitution during circulation in the bat colony (Banyai et al., 2017). We identified the common conserved sequences at the 5’ and 3’ ends of different segments, which are common genomic features for rotavirus J.

We found a pestivirus-like virus in library CS10, while library CS9 was negative for this newly identified virus (Gao et al., 2020). Comparison with known pestivirus species showed amino acid substitutions between the newly identified strain Flavivirus-YN-CHN-2020 and two DYPV strains, resulting in an emerging lineage in the phylogenetic tree. Non-synonymous substitutions appear to have accumulated in these pestivirus-like viruses, revealing their rapid evolution. Pestivirus species have been identified in several hosts, including bats, pigs, rats, dolphins, cows, and other mammals, suggesting their wide distribution in a variety of hosts. Pestiviruses are known to cause haemorrhagic syndromes, abortions, and a fatal mucosal disease in mammals, which could have significant economic impacts in the breeding industry (Valdazo-Gonzalez et al., 2007; shi et al., 2016b; Blome et al., 2017). In addition, a pestivirus-like virus has been recently identified in pangolins, named DYPV, which causes hemorrhaging and skin lesions, and even death (Gao et al., 2020).

Two caliciviruses, belonging to different genera of *Caliciviridae*, were detected. Although they were in the same family, their genomic organizations were different. The amino acid divergence between strain CS9-Cali-YN-CHN-2020 and other known species was 53.9–81.8%, and it formed a single lineage in the maximum likelihood phylogenetic tree. These results suggest that it could represent a new species of *Sapovirus*, in accordance with the demarcation criteria of the ICTV (Vinje et al., 2019). Caliciviruses are traditionally recognized as pathogens infecting numerous organisms, such as humans, cattle, pigs, cats, chickens, and amphibians (Wilhelmi et al., 2003; Mor et al., 2017; Vinje et al., 2019). Some caliciviruses can cause diseases, such as feline calicivirus, which causes a respiratory disease; rabbit haemorrhagic disease virus, which causes often-fatal hemorrhaging; and Norwalk viruses, which cause gastroenteritis (Desselberger, 2019; Vinje et al., 2019). In this study, we firstly identified a calicivirus in a pangolin specimen, which shared the highest polyprotein identity with a bat calicivirus although it showed substantial genomic divergence. These results reveal the long-scale evolution of caliciviruses in bats and pangolins.

Among the newly identified RNA viruses recently reported, picornaviruses make up a higher proportion within the virosphere (Paez-Espino et al., 2016; Shi et al., 2016a,2018a). In this study, picornaviruses were identified in almost all libraries, revealing their wide distribution in nature. The detected iflavirus shared only 55.73% polyprotein identity with closest neighboring strain Rondonia_BR_15, which was collected from ticks. They shared a similar genomic organization and formed a single lineage in the phylogenetic tree. Due to the lack of demarcation criteria for *Iflaviridae* from the ICTV, the identity of numerous iflaviruses was undetermined until now (Valles et al., 2017). Based on the phylogenetic analysis and genomic organization, the strain identified in this study might be a new species or even a new genus of Iflaviridae. According to the literature, insects are the primary hosts for *Iflaviridae* (Valles et al., 2017; Wu et al., 2019). However, *Iflaviridae*-positive specimens were collected from bats, which need to be further examined for evidence of infection. We also identified two Kobuviruses or *Kobuvirus*-related viruses, that were highly abundant in the libraries. One was near the genus *Kobuvirus* in the phylogenetic tree, and the other was clustered with AiV-A10 strains. Although they neighbored or belonged to the genus *Kobuvirus*, comparison with their closest phylogenetic neighbors showed genetic divergence. Kobuviruses are associated with gastroenteritis in humans, cattle, pigs, dogs, and other animals, whereas bat *Kobuvirus*-related viruses are less documented (Tapparel et al., 2013; Valles et al., 2017; Zell et al., 2017). Enteroviruses are associated with several human diseases, such as hand foot and mouth disease (HFMD), acute flaccid paralysis (AFP), aseptic meningitis, myocarditis, and respiratory infections (Kelly et al., 2013; Xing et al., 2014). Several serotypes, such as enterovirus A71 (EV-A71), coxsackievirus A16 (CV-A16), and coxsackievirus A6 (CV-A6) have played important roles in HFMD outbreaks in the Asian-Pacific region (Zhang et al., 2011; Xing et al., 2014; Han et al., 2020a). However, the newly identified serotypes and the serotypes circulating in non-human primates have been rarely reported, especially the enteroviruses in simians, which have the potential for spill over to humans (Sadeuh-Mba et al., 2014; Han et al., 2021b). In this study, EV-A122 was detected in simian specimens, and recombination events were confirmed. Our results revealed the evolutionary characteristics of enteroviruses and provided basic data for enterovirus surveillance, especially simian enteroviruses. Posa-like viruses have a complex phylogenetic relationship and a long evolutionary time scale (Hause et al., 2016; Oude Munnink et al., 2017; Han et al., 2020b,2021a). These viruses have a wide host distribution that include both vertebrates and invertebrates, which agrees with the abundant distribution of picornavirus in nature (Shi et al., 2016a,2018a).

## Conclusion

We performed a mNGS analysis of the viromes of several host species and identified many RNA viruses in libraries generated from these specimens, including alphacoronaviruses from bat specimens. Some of them showed higher divergence from known virus species, in terms of their genome characteristics and organization. Moreover, we also characterized the evolutionary dynamics of these new viruses by comparison to known families, revealing their host distribution. Furthermore, some of the novel viruses discovered in this study, could be assigned to new species, and even new genera. Taken together, this study provides valuable basic data for RNA viruses of different families, which can improve our understanding of the virome. However, only specimens from the Yunnan province were collected in this study, although these specimens were collected from several host species, which could represent the viromes in local regions. More specimens from other regions in China, even countries, warrant further investigation for revealing the virome changes. Some novel RNA viruses, e.g., Ifla-YN-CHN-2020, was not classified till now, because the ICTV lack a clear demarcation criteria for *Iflaviridae*. The condition hindered the taxonomical assignment, such as a new species or even a new genus of *Iflaviridae*. It awaited a more comprehensive viral classification system under the metagenomic age nowadays. The data document of novel viral evolution, recombination, even spill over perfect the requirement for viral investigation, as revealed by this study.

## Data availability statement

The data presented in this study have been deposited in GenBank under accession numbers MT649088, MT649091, MT878534, MT878532, and MW450824–MW450843. The meta-transcriptomics sequencing data were submitted to the NCBI Sequence Read Archive (SRA) under accession numbers SRP270853 and PRJNA689958.

## Ethics statement

The studies involving human participants were reviewed and approved the Second Ethics Review Committee of the National Institute for Viral Diseases Control and Prevention (IVDC) and Chinese Center for Diseases Control and Prevention. The patients/participants provided their written informed consent to participate in this study. All necropsy and sample collection procedures were performed in strict accordance with the China CDC guidelines for the Laboratory Animal Use and Care [SYXK(Jing)2017-0021]. The study was also approved by the Animal Ethics Review Committee of the National Institute for Viral Diseases Control and Prevention (IVDC) and Chinese Center for Diseases Control and Prevention.

## Author contributions

ZH conceived and performed the experiments, analyzed the data, drafted the manuscript, and prepared all the figures. YZ, XF, and WX conceived and designed the experiments, supervised the project, and polished the manuscript. JX, YS, XZ, QS, HL, KZ, JcL, JhL, FS, GZ, HZ, SJ, and JZ conducted some of the experiments. DW, SZ, and DY analyzed the data. All authors reviewed and approved the final manuscript.

## Supplementary material

Supplementary Figure 1 Genomic organization and similarity plots of two representative coronavirus strains identified in this study, strain ATG20-YN-CHN-2020 **(A)** and strain ATG32-YN-CHN-2020. The phylogenetic neighbors were selected for the similarity plot. The black line indicates 90% similarity.

Supplementary Figure 2 Maximum likelihood tree based on the RNA-dependent RNA polymerase (RdRp) core protein sequences of the reference genomes from eight families of Picornavirales and a newly identified picornavirus. The scale bars show the substitutions per site per year, and the values at each node indicate the percent of SH-like approximate likelihood ratio tests (SH-aLRT), with 1,000 bootstrap replicates. The black arrows represent the partial genomes detected in this study. The family Caliciviridae was used as an outgroup.

Supplementary Figure 3 Genomic organization, phylogenetic analysis, and potential recombination evens in the newly identified mamastrovirus strains. **(A)** Genomic organization of newly identified strains. **(B)** Maximum likelihood tree of mamastrovirus reference genomes. The black arrows represent the newly identified strains. The scale bars show the substitutions per site per year, and the values at each node indicate the bootstrap and SH-like approximate likelihood ratio tests (SH-aLRT), with 1,000 bootstrap replicates. **(C)** The inferred recombination events in the newly identified mamastrovirus strain.

Supplementary Figure 4 Maximum likelihood phylogenetic trees of the newly identified mamastrovirus strains and phylogenetic neighboring mamastroviruses, based on the amino acid sequences of **(A)** ORF1a, **(B)** ORF1b, and **(C)** ORF2. The scale bars show the number of substitutions per site per year, and the black arrows represent the newly identified strains. The values at each node indicate the bootstrap and SH-like approximate likelihood ratio tests (SH-aLRT), with 1,000 bootstrap replicates.

Supplementary Figure 5 Maximum likelihood phylogenetic trees of different rotavirus species and newly identified bat rotaviruses, which were based on the reference rotavirus genome sequences of NSP1 **(A)**, NSP2 **(B)**, NSP3 **(C)**, NSP4 **(D)**, NSP5 **(E)**, VP1 **(F)**, VP2 **(G)**, VP3 **(H)**, and VP6 **(I)**. The black arrows represent the newly identified strains. The scale bars show the substitutions per site per year. The values at each node indicate the bootstrap and SH-like approximate likelihood ratio tests (SH-aLRT), with 1,000 bootstrap replicates.

Supplementary Figure 6 Characterization of the rotavirus genome contigs. **(A)** The top two hits in the nucleotide (NT) and protein (NR) databases obtained using BLASTN and BLASTP, based on the VP6 segment of a newly identified rotavirus. **(B)** Assignment and characteristics of the genome segments of a newly identified bat rotavirus. Maximum likelihood trees of different rotavirus species and bat rotaviruses based on the amino acid sequences of VP6 **(C)** and VP1 **(D)** segments. The black arrows represent the strains identified in this study. Each color module represents a different rotavirus species. The scale bars show the substitutions per site per year. The values at each node indicate the bootstrap and SH-like approximate likelihood ratio test (SH-aLRT), with 1,000 bootstrap replicates.

Supplementary Figure 7 Characterization of the calicivirus genome contigs. **(A)** Genomic organization of calicivirus and annotated conserved domains. **(B)** Maximum likelihood tree of the complete VP1 amino acid sequences, including the reference genomes of all *Caliciviridae* and the two newly identified strains in this study. The scale bars show the substitutions per site per year, and the values at each node indicate the bootstrap and SH-like approximate likelihood ratio tests (SH-aLRT), with 1,000 bootstrap replicates. The black arrows represent the newly identified strains.

Supplementary Figure 8 **(A)** Genomic organization of caliciviruses and annotated conserved domains. **(B)** Maximum likelihood tree of the full-length polyprotein sequences of neighboring genomes and the newly identified strain. The scale bars show the substitutions per site per year, and the values at each node indicate the bootstrap and SH-like approximate likelihood ratio tests (SH-aLRT), with 1,000 bootstrap replicates. The black arrows represent the newly identified strains.

Supplementary Figure 9 **(A)** Genomic organization of kobuvirus-related viruses and annotated conserved domains. **(B)** The top two hits in the nucleotide (NT) and protein (NR) databases using BLASTN and BLASTP. **(C)** Maximum likelihood tree of the complete amino acid sequences of the reference genomes of known species of kobuvirus and neighboring strains. The scale bars show the substitutions per site per year, and the values at each node indicate the bootstrap and SH-like approximate likelihood ratio tests (SH-aLRT), with 1,000 bootstrap replicates. The black arrows represent the newly identified strains.

Supplementary Figure 10 **(A)** Genomic organization of the newly identified kobuvirus in this study and the mapped region. **(B)** The top two hits in the nucleotide (NT) and protein (NR) database using BLASTN and BLASTP. **(C)** Maximum likelihood tree of the complete P1 coding region sequences, including the reference genomes of known species of kobuvirus and the newly identified strain. The scale bars show the substitutions per site per year, and the values at each node indicate the bootstrap and SH-like approximate likelihood ratio tests (SH-aLRT), with 1,000 bootstrap replicates. The black arrows represent the newly identified strains.

Supplementary Figure 11 **(A)** Genomic organization of the newly identified EV-A122 strains in this study. Maximum likelihood tree of the complete P1 **(B)** and P3 **(C)** coding region sequences, including the reference genomes of known serotypes of enterovirus A and the newly identified strains. The scale bars show the substitutions per site per year, and the values at each node indicate the bootstrap and SH-like approximate likelihood ratio tests (SH-aLRT), with 1,000 bootstrap replicates. The black arrows represent the newly identified strains. **(D)** Similarity plot of the circulating recombination donors and newly identified strains. The genomic map (upper) and recombination events predicted for the strains in this study, which is shown as a black block. Genetic components identified by RDP4, which were involved in the recombination events, are shown as different color blocks. Likely breakpoint positions are shown above the genome.

Supplementary Figure 12 Characterization of the posa-like virus genome contigs. **(A)** Genomic organization of posa-like viruses and annotated conserved domains. **(B)** Maximum likelihood tree of the complete amino acid sequences, including the reference genomes of known species of posa-like virus and neighboring strains. The scale bars show the substitutions per site per year, and the values at each node indicate the bootstrap and SH-like approximate likelihood ratio tests (SH-aLRT), with 1,000 bootstrap replicates. The black arrows represent the newly identified strains.

Supplementary Table 1 List of host species, their geographic distributions, and the collection dates for the samples analyzed in this study.

Supplementary Table 2 The geographic distribution, species information, and data statistics for each library. All counties or cities were located in Yunnan province, China. The output data were calculated based on the raw reads of each library.

Supplementary Table 3 Primers and probes used for viral identification.
