# Supplementary material for: Highly diverse ribonucleic acid viruses in the viromes of eukaryotic host species in Yunnan province, China
Source: Front Microbiol. 2022 Oct 13;13:1019444. doi: 10.3389/fmicb.2022.1019444 (PMC9606678; doi:10.3389/fmicb.2022.1019444)

Supplementary materials

Supplementary content for maintext

Mamastroviruses

Mamastroviruses were detected in three libraries (BF1, BF2, and BF3), and they were present in a large number of samples (Table 2). The assembled contigs shared 76%–82% genomic identity with known mamastrovirus strains. We obtained one nearly full-length genome sequence, BF2-astro-YN-CHN-2020. It clustered with Mamastrovirus-18-isolate-AFCD337 (GenBank accession no. NC043102), with high bootstrap support (S3 Fig). Analysis of the BF2-astro-YN-CHN-2020 genome showed that it contained three ORFs, ORF1a, ORF1b, and ORF2, respectively (S3A Fig), encoding a viral trypsin-like serine protease, RNA-dependent RNA polymerase (RdRp), and capsid precursor protein, respectively, which is similar to the genetic organization typical of other mamastroviruses. To assess whether the genomic sequence could be related to sequences of viruses infecting other hosts (e.g. humans, cows, dogs, marmots, and cats), a phylogenetic analysis was performed based on the predicted amino acid sequences of three ORFs from different hosts (S4 Fig). All three ORFs clustered with bat astroviruses in the phylogenetic tree, although ORF2 was located in the ancestral node of the phylogenetic tree, with a bootstrap value of 100% (S4C Fig). This suggested that a recombination event may have occurred in the ORF2 region in a bat and is still circulating and spreading in the bat population (S3C Fig and S4C Fig). This result

illustrates the significant genomic variation in mamastroviruses compared to known strains, revealing ongoing genetic changes in the bat population.

Rotaviruses

We identified seven rotaviruses in three bat libraries (BF1, BF2, and BF3), and each library contained several contigs (Table 2). Some de novo contigs shared ~94% genomic identity with strain BO4351/Ms/2014 (GenBank accession no. KX756624.1), which was collected in Serbia in October 2014. After manual inspection and assembly, we harvested nine nearly full-length segments of rotavirus. However, two segments, VP4 and VP7, were not identified in this study, despite conducting qRT-PCR assays targeting these two segments (S3 Table). The results showed that all segments of Rota-BF-YN-CHN-2020 were clustered with a known strain, BO4351/Ms/2014, in the phylogenetic tree, which is assigned as a rotavirus J species, with bootstrap values of 100% for each segment (S5 Fig). Based on the protein sequences of VP6 and VP1, the closest phylogenetic neighbour of Rota-BF-YN-CHN-2020 was strain BO4351/Ms/2014, and we did not find any other strains that shared higher genomic identity with Rota-BF-YN-CHN-2020 (others had <50% amino acid identity in VP6, and lower amino acid identity in other segments), except for strain BO4351/Ms/2014 (S6 Fig). The 5′ end of the genomic segments contained a conserved GAAA sequence, while the 3′ ends contained a conserved sequence of AATAYACCC (S6B Fig). The results revealed that strain Rota-BF-YN-CHN-2020 was a rotavirus J species, and only one known strain had a similar phylogeny. This is the first report of a rotavirus J species in China.

Caliciviruses

We identified one calicivirus each in the CS9 and YN19 libraries (Table 2). These two caliciviruses were highly abundant in the mNGS data, and we obtained nearly full-length genomes through manual inspection. The viruses shared about 66% and 94% genomic identity with known genomes. Strain YN19-Cali-YN-CHN-2020 contained three ORFs (ORF1–3), which encoded a non-structural protein, capsid precursor protein 1, and minor capsid protein 2, respectively (S7A Fig). Some conserved domains were annotated, including the helicase-peptidase-RdRp domain in ORF1 and the calici-coat-like domain in ORF2. The genomic organization of YN19-Cali-YN-CHN-2020 was similar to that of other strains of the genus *Vesivirus* (Vinje et al., 2019). Based on the maximum likelihood tree of the complete VP1 amino acid sequences, strain YN19-Cali-YN-CHN-2020 clustered with the genus *Vesivirus* and showed high similarity to a mink calicivirus (GenBank accession no. MF677852) (S7B Fig). Based on the complete amino acid sequences of VP1, another strain, CS9-Cali-YN-CHN-2020, clustered within the genus *Sapovirus* but was separated into a new lineage (S7B Fig). The amino acid similarity of CS9-Cali-YN-CHN-2020 to other viruses of the genus ranged from 53.9%–81.8%, based on the complete VP1 amino acid sequences (data not shown). However, the genome of strain CS9-Cali-YN-CHN-2020 contained two ORFs (ORFs 1 and 2), which encoded a non-structural protein and capsid precursor protein 1 as well as minor capsid protein 2, respectively (S8A Fig). The genome arrangement of CS9-Cali-YN-CHN-2020 was different from that of YN19-Cali-YN-CHN-2020. Based on the full-length polyproteins of neighbouring strains, CS9-Cali-YN-CHN-2020 was closest to a bat calicivirus (GenBank accession no. KJ641701) (S8B Fig). However, the amino acid divergence of strain CS9-Cali-YN-CHN-2020 indicated that it was a new species within the

genus *Sapovirus*, as the virus only shared 66% genomic similarity with known strains, corresponding to demarcation criteria of the International Committee on Taxonomy of Viruses (ICTV). This is the first identification of a calicivirus in a specimen from pangolins (Vinje et al., 2019).

Picornaviruses

Kobuvirus and kobuvirus-like strains were detected in libraries BF1 and BF2, respectively (Table 2). The genome of BF2-picornavirus-YN-CHN-2020 contained an ORF that encoded a precursor polyprotein and was predicted to be cleaved into several functional proteins, including a non-structural protein and capsid protein (S9A Fig). The phylogenetic tree showed that BF2-picornavirus-YN-CHN-2020 was clustered with a bat unclassified picornavirus (GenBank accession no. MF352427.1), which was near the genus *Kobuvirus* in the family *Picornaviridae* (S9C Fig). The phylogenetic relationships based on the RdRp core sequence revealed that BF2-picornavirus-YN-CHN-2020 was clustered within the family *Picornaviridae*, confirming its taxonomical classification (S2 Fig). The BF1-Kobuvirus-YN-CHN-2020 partial genome, which only covered the P1 coding region, shared 86.9% genomic identity with strain 16845x64 (GenBank accession no. MF947438.1) (S10A Fig) and was clustered with Aichi virus A10 (AiV-A10) strains, with high bootstrap support. Its genome showed ~13% nucleotide divergence compared with its closest relative, strain 16845x64 (S10B–C Fig). These results, comparing BF1-Kobuvirus-YN-CHN-2020-Partial-genome to other existing kobuvirus species, suggest that it may have evolved and may represent a new genotype or genogroup within the AiV-A10.

We identified a simian enterovirus 19 (EV-A122) and monsavirus (posa-like virus) in two libraries, MO13 and MO14, which were present in high abundance (Table 2). Nine and thirteen specimens were positive for EV-A122 and posa-like viruses, respectively. The full-length genome of EV-A122 was obtained, and its genomic organization was typical of enteroviruses (S11A Fig), encoding a polyprotein that was predicted to be cleaved into a structural protein (capsid) and two non-structural proteins. The phylogenetic tree based on the *P1* coding region revealed that the strains detected in this study (AKM5-YN-CHN-2020 and JH05-YN-CHN-2020) clustered with the prototype strain EV-A122 (GenBank accession no. AF326754.2), whereas, in the tree based on the P3 coding region, they clustered with the prototype strain EV-A92 (GenBank accession no. EF667344.1) (S11B–C Fig). The results suggested that strains AKM5-YN-CHN-2020 and JH05-YN-CHN-2020 belonged to the EV-A122 genotype and that recombination events occurred in the P2 and P3 coding regions, with CHN/BJ/2018-1A (GenBank no. MN427525.1) inferred as a recombination parent, based a search for circulating potential donors (S11D Fig). These results were in agreement with reports of frequent recombination events among enteroviruses (Lukashev, 2005;Geoghegan et al., 2015;Kyriakopoulou et al., 2015;Tan et al., 2016;Han et al., 2018).

Posa-like viruses contain a polyprotein that is cleaved into capsid and non-structural proteins. We detected four conserved domains, which covered both the non-structural and structural regions (S12 Fig). Posa-like viruses have a different genomic organization compared to that of EV-A122, specifically the genomic location of structural protein-encoding region. Based on the phylogeny of the RdRp core domain, the strains identified in this study were clustered with other posa-like viruses, which has been proposed as a family within the *Picornavirus* (S2 Fig) (Zhang et al.,

2014;Oude Munnink et al., 2017;Aoki et al., 2019;Han et al., 2020;Han et al., 2021). A phylogenetic tree based on the complete amino acid sequences revealed that these strains clustered with strain Tottori-HG1 (GenBank accession no. LC123275.1), which was identified in the host *Sus scrofa domesticus* in Japan (S12B Fig). These results suggest a comprehensive host distribution and complex phylogenetic relationships among posa-like viruses.

Reference

Aoki, H., Sunaga, F., Ochiai, H., Masuda, T., Ito, M., Akagami, M., Naoi, Y., Sano, K., Katayama, Y., Omatsu, T., Oba, M., Sakaguchi, S., Furuya, T., Ouchi, Y., Shirai, J., Mizutani, T., Oka, T., and Nagai, M. (2019). Phylogenetic analysis of novel posaviruses detected in feces of Japanese pigs with posaviruses and posa-like viruses of vertebrates and invertebrates. *Arch Virol* 164, 2147-2151.

Geoghegan, J.L., Tan Le, V., Kuhnert, D., Halpin, R.A., Lin, X., Simenauer, A., Akopov, A., Das, S.R., Stockwell, T.B., Shrivastava, S., Ngoc, N.M., Uyen Le, T.T., Tuyen, N.T., Thanh, T.T., Hang, V.T., Qui, P.T., Hung, N.T., Khanh, T.H., Thinh Le, Q., Nhan Le, N.T., Van, H.M., Viet Do, C., Tuan, H.M., Viet, H.L., Hien, T.T., Chau, N.V., Thwaites, G., Grenfell, B.T., Stadler, T., Wentworth, D.E., Holmes, E.C., and Van Doorn, H.R. (2015). Phylodynamics of Enterovirus A71-Associated Hand, Foot, and Mouth Disease in Viet Nam. *J Virol* 89, 8871-8879.

Han, Z., Song, Y., Xiao, J., Zhao, X., Lu, H., Zhang, K., Jia, S., Zhou, J., Li, J., Si, F., Sun, Q., Zhu, S., Wang, D., Yan, D., Xu, W., Fu, X., and Zhang, Y. (2021). Monsavirus in monkey rectal swab and throat swab specimens in China: Proposal for Posaliviridae as a new family in Picornavirales. *Virus Res*, 198501.

Han, Z., Xiao, J., Song, Y., Hong, M., Dai, G., Lu, H., Zhang, M., Liang, Y., Yan, D., Zhu, S., Xu, W., and Zhang, Y. (2020). The Husavirus Posa-Like Viruses in China, and a New Group of Picornavirales. *Viruses* 12.

Han, Z., Zhang, Y., Huang, K., Cui, H., Hong, M., Tang, H., Song, Y., Yang, Q., Zhu, S., Yan, D., and Xu, W. (2018). Genetic characterization and molecular epidemiological analysis of novel enterovirus EV-B80 in China. *Emerg Microbes Infect* 7, 193.

Kyriakopoulou, Z., Pliaka, V., Amoutzias, G.D., and Markoulatos, P. (2015). Recombination among human non-polio enteroviruses: implications for epidemiology and evolution. *Virus Genes* 50, 177-188.

Lukashev, A.N. (2005). Role of recombination in evolution of enteroviruses. *Rev Med Virol* 15, 157-167.

Oude Munnink, B.B., Phan, M.V.T., Consortium, V., Simmonds, P., Koopmans, M.P.G., Kellam, P., Van Der Hoek, L., and Cotten, M. (2017). Characterization of Posa and Posa-like virus genomes in fecal samples from humans, pigs, rats, and bats collected from a single location in Vietnam. *Virus Evol* 3**,** vex022.

Tan, Y., Hassan, F., Schuster, J.E., Simenauer, A., Selvarangan, R., Halpin, R.A., Lin, X., Fedorova, N., Stockwell, T.B., Lam, T.T., Chappell, J.D., Hartert, T.V., Holmes, E.C., and Das, S.R. (2016). Molecular Evolution and Intraclade Recombination of Enterovirus D68 during the 2014 Outbreak in the United States. *J Virol* 90**,** 1997-2007.

Vinje, J., Estes, M.K., Esteves, P., Green, K.Y., Katayama, K., Knowles, N.J., L'homme, Y., Martella, V., Vennema, H., White, P.A., and Ictv Report, C. (2019). ICTV Virus Taxonomy Profile: Caliciviridae. *J Gen Virol* 100**,** 1469-1470.

Zhang, B., Tang, C., Yue, H., Ren, Y., and Song, Z. (2014). Viral metagenomics analysis demonstrates the diversity of viral flora in piglet diarrhoeic faeces in China. *J Gen Virol* 95**,** 1603-1611.

Table S1. List of host species, their geographic distributions, and the collection dates for the samples analysed in this study.

Region	Species	Collection date	Sample type*	Sample number
Mojiang county	*Rhinolophus sinicus*	2020/02/19-2020/02/22	Throat swabs and anal swab	120
	Miniopterus schreibersii		Throat swabs and anal swab	50
	Rhinolophus pusillus		Throat swabs and anal swab	4
	Rhizomys		Throat swabs and anal swab	22
	Paguma larvata		Throat swabs and anal swab	6
	Rhesus macaque		Throat swabs and anal swab	8
	Macaca assamensis		Throat swabs and anal swab	4
	Nycticebus pygmaeus		Throat swabs and anal swab	2
	Callosciurus erythraeus		Throat swabs and anal swab	4
	Homo sapiens		Throat swabs and anal swab	12
	Hystrix brachyura hodgsoni		Throat swabs and anal swab	20
Dehong county	*Malayan pangolin*	2020/2/20	Throat swabs and tissues samples†	30
	Nycticebus pygmaeus		Throat swabs and anal swab	10
	Rhesus macaque		Throat swabs and anal swab	4
	Macaca arctoides		Throat swabs and anal swab	2

Puer city	*Malayan pangolin*	2020/02/21-2020/02/23	Throat swabs and tissues samples†	22
	Paguma larvata		Throat swabs and anal swab	2
	Rhesus macaque		Throat swabs and anal swab	2
	Strix aluco		Throat swabs and anal swab	2
	Prionailurus bengalensis		Throat swabs and anal swab	2
	Nycticebus pygmaeus		Throat swabs and anal swab	2
Xishuangbanna county	*Malayan pangolin*	2020/02/25-2020/02/27	Throat swabs and anal swab	4
	Homo sapiens		Throat swabs and serum samples	4
	Rhesus macaque		Throat swabs and anal swab	4
	Prionailurus bengalensis		Throat swabs and anal swab	4
	Macaca leonina		Throat swabs and anal swab	4
Baoshan city	*Ailurus fulgens*	2020/2/28	Throat swabs and anal swab	10
	Homo sapiens		Throat swabs and serum samples	12
	Macaca thibetana		Throat swabs and anal swab	8
	Vicugna pacos		Throat swabs and anal swab	4
	Vulpes		Throat swabs and anal swab	8
Tengchong city	*Manis pentadactyla*	2020/02/25-2020/02/27	Throat swabs and tissues samples†	29

	Homo sapiens		Throat swabs and serum samples	8
	Vulpes		Throat swabs and anal swab	8
Kunming city	*Rhesus macaque*	2020/2/23	Throat swabs and anal swab	14
	Macaca thibetana		Throat swabs and anal swab	2
	Macaca nemestrina		Throat swabs and anal swab	4
	Paguma larvata		Throat swabs and anal swab	2
	Vulpes		Throat swabs and anal swab	4
	Procyon lotor		Throat swabs and anal swab	2

* The samples collected from Homo sapiens were the breeders or human contacts of animals.

† The tissues samples contain the lung, heart, spleen, intestines and muscle of animals.

Table S2. The geographic distribution, species information, and data statistics for each library. All counties or cities were located in Yunnan province, China. The output data were calculated based on the raw reads of each library.

Pool	Number of units	Species	Region	Data (reads)
BF1	23	*Rhinolophus sinicus*<br>*Miniopterus schreibersii*	Mojiang county	177182008
BF2	23	*Rhinolophus sinicus*<br>*Miniopterus schreibersii*	Mojiang county	162981144
BF3	22	*Rhinolophus sinicus*<br>*Miniopterus schreibersii*	Mojiang county	193887774
CS9	20	*Malayan pangolin*<br>*Manis pentadactyla*	Xishuangbanna county; Puer city; Baoshan city	168460686
CS10	28	*Malayan pangolin*	Dehong county	368617768
Mo13	32	*Rhesus macaque; Macaca thibetana; Macaca nemestrina*	Kunming city; Mojiang county	172681140
Mo14	28	*Rhesus macaque; Macaca nemestrina*	Baoshan city; Xishuangbanna county	176787228
YN19	19	*Paguma larvata; Callosciurus erythraeus; Prionailurus bengalensis; Mephitis*	Tengchong city; Xishuangbanna county; Dehong county; Mojiang county	303839282

Table S3. Primers and probes used for viral identification.

Primer	Viruses type*	Primer sequence (5'-3')	Orientation
Kobuvirus-1670-P-F	Kobuvirus	CCC AAA CTC AGA CRC AGG ACC TGG	Forward
Kobuvirus-1770-P-R		GGG TGG GGG TTC TCA GTG TAA TCG	Reverse
Kobuvirus-1730-Probe		ROX-ATG GTG CCG CGG AYA ACA CCC CGC-BHQ2	
Iflavirus-3210-P-F	Iflavirus	AGGCGCGACGTTTATGCTCGGTCG	Forward
Iflavirus-3340-P-R		CAATGGGTCAGGATAACCGTTACG	Reverse
Iflavirus-3280-P-Probe		FAM-CAA TGC GCG TTC CGT TTA AAG CAT-BHQ1	
Mamastrovirus-3100-P-F	Mamastrovirus	GATTTTGATGCGCTCGTGGGAAAG	Forward
Mamastrovirus-3230-P-R		GAGTGGATCCCCACCACTAACGCG	Reverse
Mamastrovirus-3150-Probe		FAM-CACATAATTTGCCGGATGACGATC-BHQ1	
Dicistroviridae-5420-P-F	Dicistroviridae	GAAAGATGAGCGTCGACCCATTGA	Forward
Dicistroviridae-5640-P-R		ATCTCCAGCAATTACTTTTTCTCC	Reverse
Dicistroviridae-5490-Probe		ROX-GGACCAATGGACTTTTCTATAGCT-BHQ2	
Rotavirus-2470(VP1)-P-F	Rotavirus VP1 gene	CGCCGGTTACATTGTTCGCAACGG	Forward
Rotavirus-2470(VP1)-P-R		CTGGTTGAACTGAGATTGACAGAGATG	Reverse

Rotavirus-2530(VP1)-Probe		FAM-GGAAGAATGAGATTGTACTCAGAC-BHQ1	
Rotavirus-1245(VP4)-P-F	Rotavirus VP4 gene	CGGTGGGTTTGGACAGATTGACTC	Forward
Rotavirus-1401(VP4)-P-R		GATGGGTCCTCTCTCCATGCTGGC	Reverse
Rotavirus-1321(VP4)-Probe		ROX-CCAGAGTATAAGACTGCTTGGGAA-BHQ2	
Rotavirus-121(VP7)-P-F	Rotavirus VP7 gene	GCGGATAAAATAAATAATCTTCTCGAC	Forward
Rotavirus-281(VP7)-P-R		CGTTTCCAAGCAAAGTGGCGAGATCCG	Reverse
Rotavirus-201(VP7)-Probe		CY5-GGATGCCATAGATGCGATTTCAAAATG-BHQ3	
Caliciviridae-CS9-3320-P-F	Caliciviridae	GGTGTGTTCGATGCAGATGGAGCC	Forward
Caliciviridae-CS9-3420-P-R		GGATAGGCAGGGCCTTTGTATTTGGTC	Reverse
Caliciviridae-CS9-3390-Probe		FAM-GCTGCCCACTTGTTCAGTGATGGA-BHQ1	
Flaviviridae-CS10-170-P-F	Flaviviridae	CTGATAGGGTATCGGCGCCGATGG	Forward
Flaviviridae-CS10-320-P-R		GGTGTAACTCACCGTGTTTCACAC	Reverse
Flaviviridae-CS10-250-Probe		FAM-GAGTAGGGACAACACCACCAAGAG-BHQ1	

Fig S1. Genomic organization and similarity plots of two representative coronavirus strains identified in this study, strain ATG20-YN-CHN-2020 (A) and strain ATG32-YN-CHN-2020. The phylogenetic neighbours were selected for the similarity plot. The black line indicates 90% similarity.

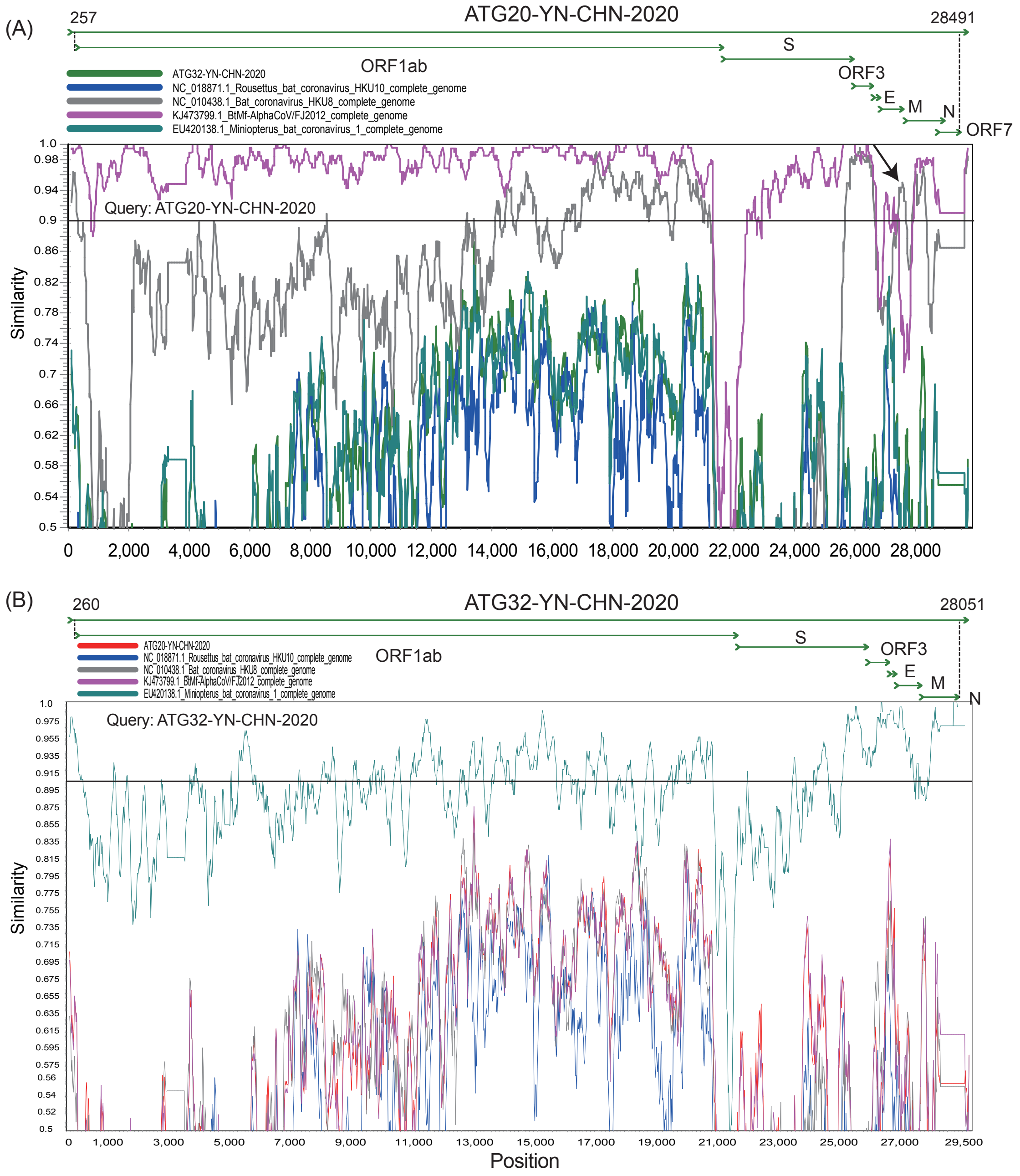

Fig S2. Maximum likelihood tree based on the RdRp core protein sequences of the reference genomes from eight families of Picornavirales and a newly identified picornavirus. The scale bars show the substitutions per site per year, and the values at each node indicate the percent of SH-like approximate likelihood ratio tests (SH-aLRT), with 1000 bootstrap replicates. The black arrows represent the partial genomes detected in this study. The family Caliciviridae was used as an outgroup.

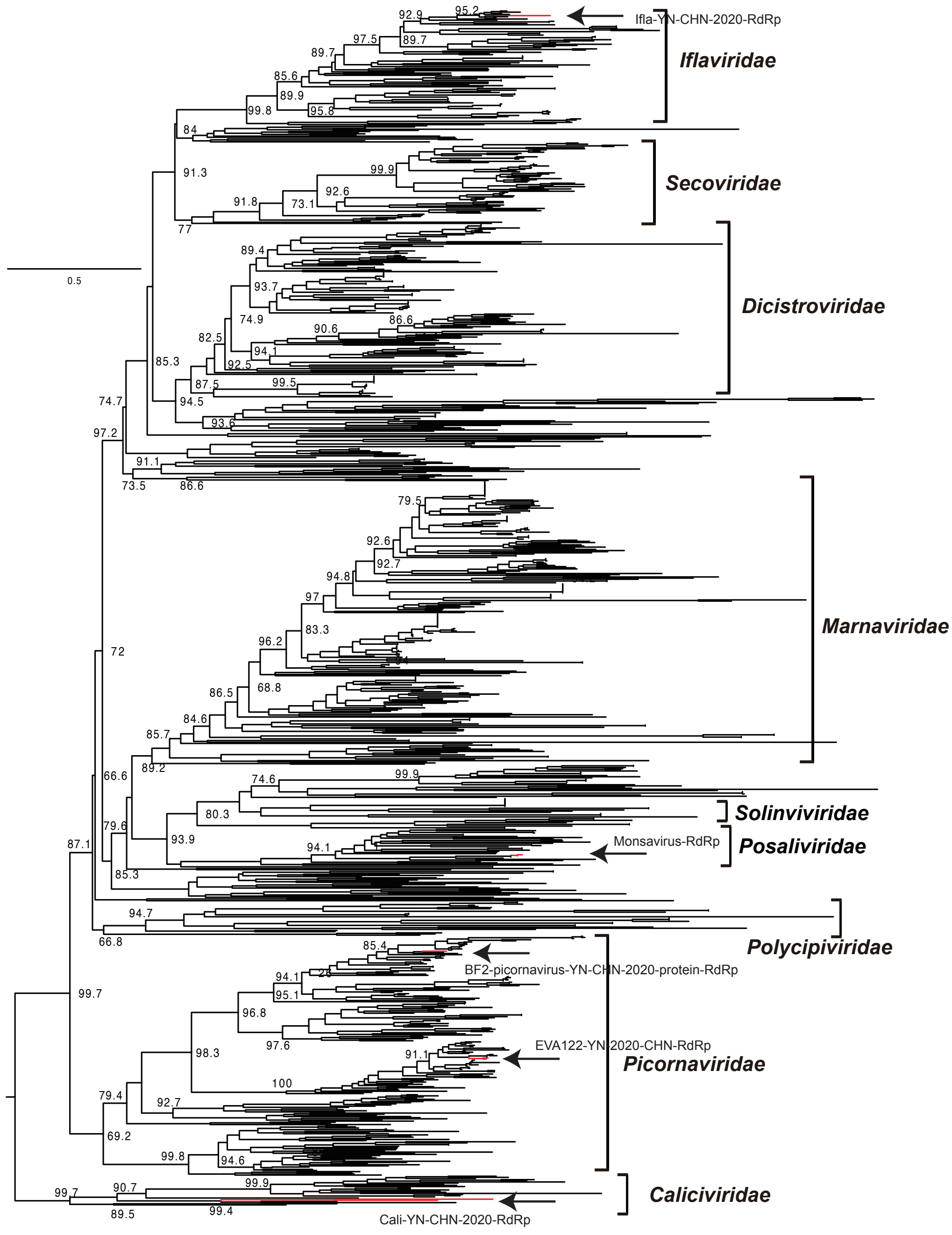

Fig S3. Genomic organization, phylogenetic analysis, and potential recombination evens in the newly identified mamastrovirus strains. (A) Genomic organization of newly identified strains. (B) Maximum likelihood tree of mamastrovirus reference genomes. The black arrows represent the newly identified strains. The scale bars show the substitutions per site per year, and the values at each node indicate the bootstrap and SH-like approximate likelihood ratio tests (SH-aLRT), with 1000 bootstrap replicates. (C) The inferred recombination events in the newly identified mamastrovirus strain.

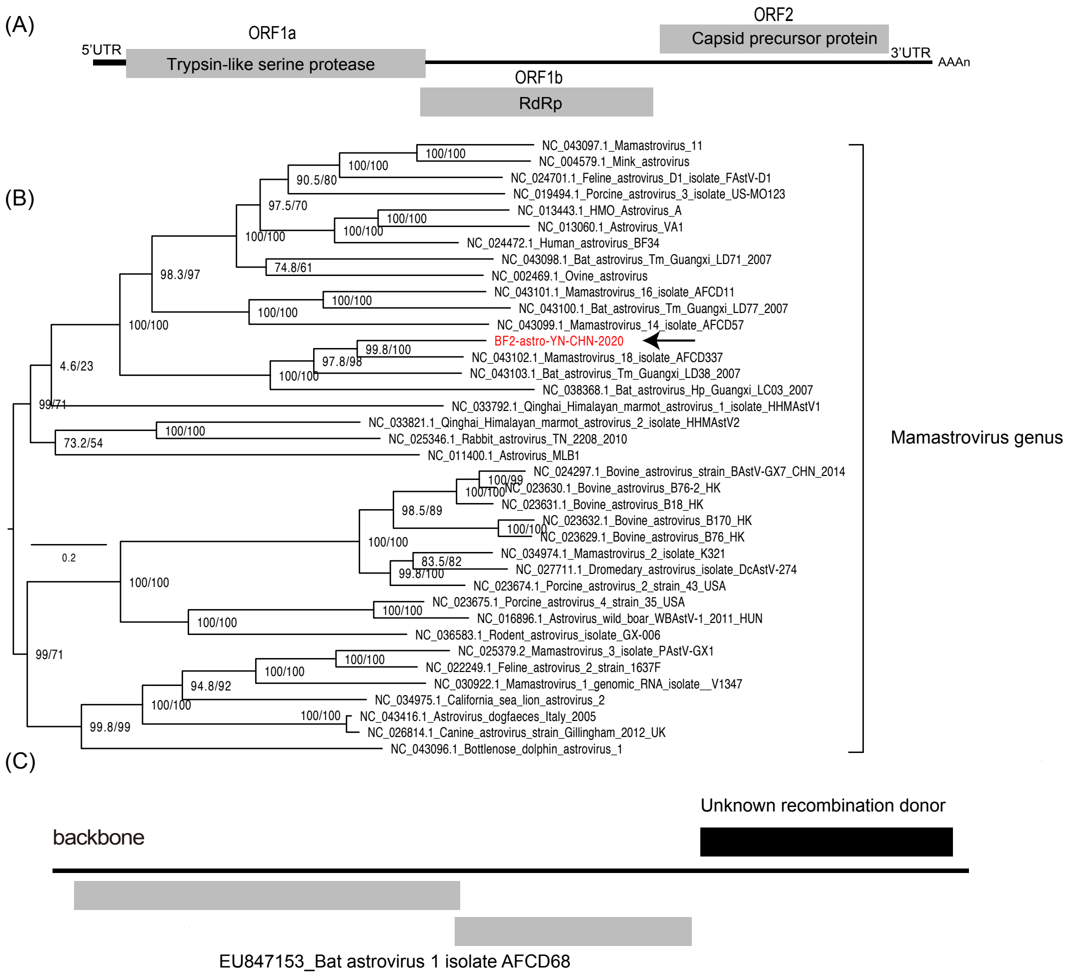

Phylogenetic tree of different genomic regions shown in Figure S2

RdRp: RNA-dependent RNA polymerase

Fig S4 Maximum likelihood phylogenetic trees of the newly identified mamastrovirus strains and phylogenetic neighbouring mamastroviruses, based on the amino acid sequences of (A) ORF1a, (B) ORF1b, and (C) ORF2. The scale bars show the number of substitutions per site per year, and the black arrows represent the newly identified strains. The values at each node indicate the bootstrap and SH-like approximate likelihood ratio tests (SH-aLRT), with 1000 bootstrap replicates.

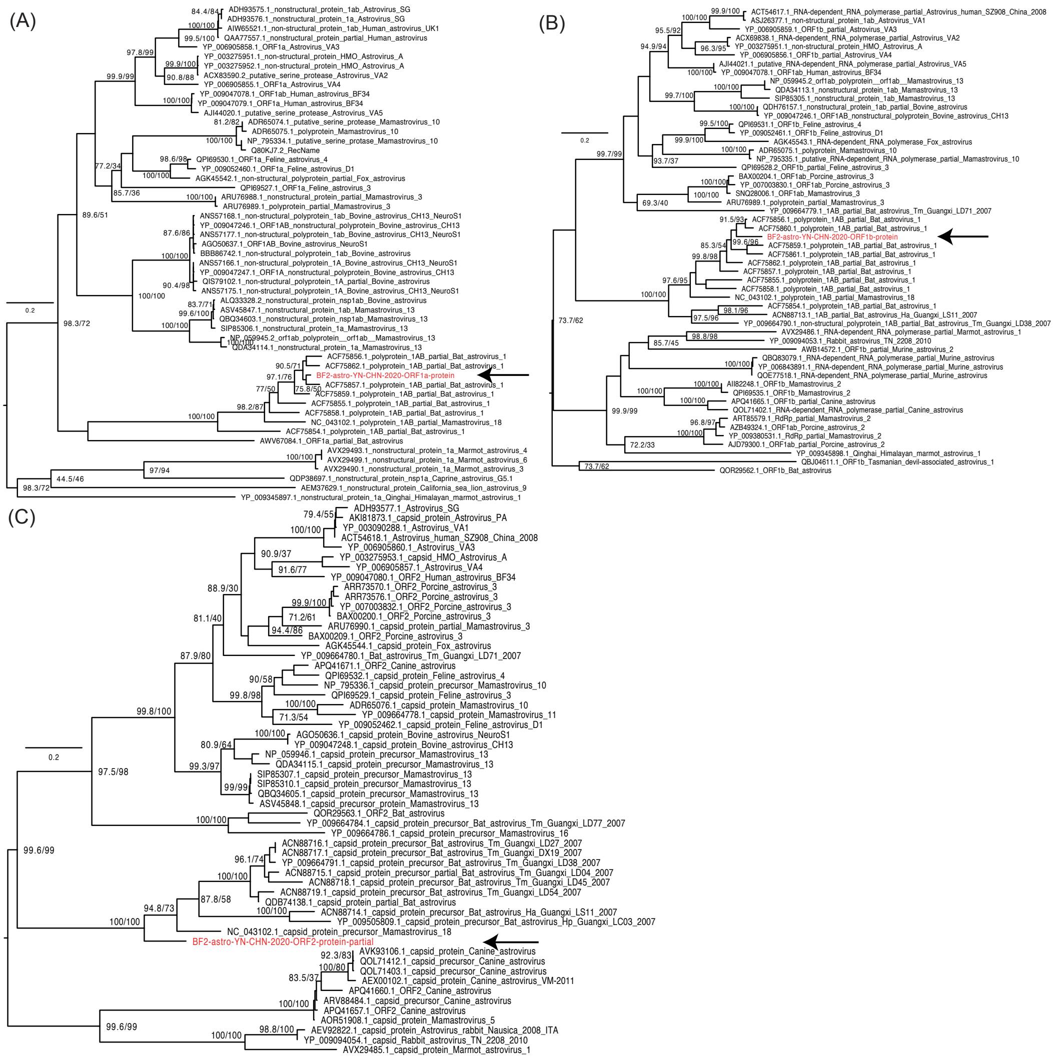

Fig S5. Maximum likelihood phylogenetic trees of different rotavirus species and newly identified bat rotaviruses, which were based on the reference rotavirus genome sequences of NSP1 (A), NSP2 (B), NSP3 (C), NSP4 (D), NSP5 (E), VP1 (F), VP2 (G), VP3 (H), and VP6 (I). The black arrows represent the newly identified strains. The scale bars show the substitutions per site per year. The values at each node indicate the bootstrap and SH-like approximate likelihood ratio tests (SH-aLRT), with 1000 bootstrap replicates.

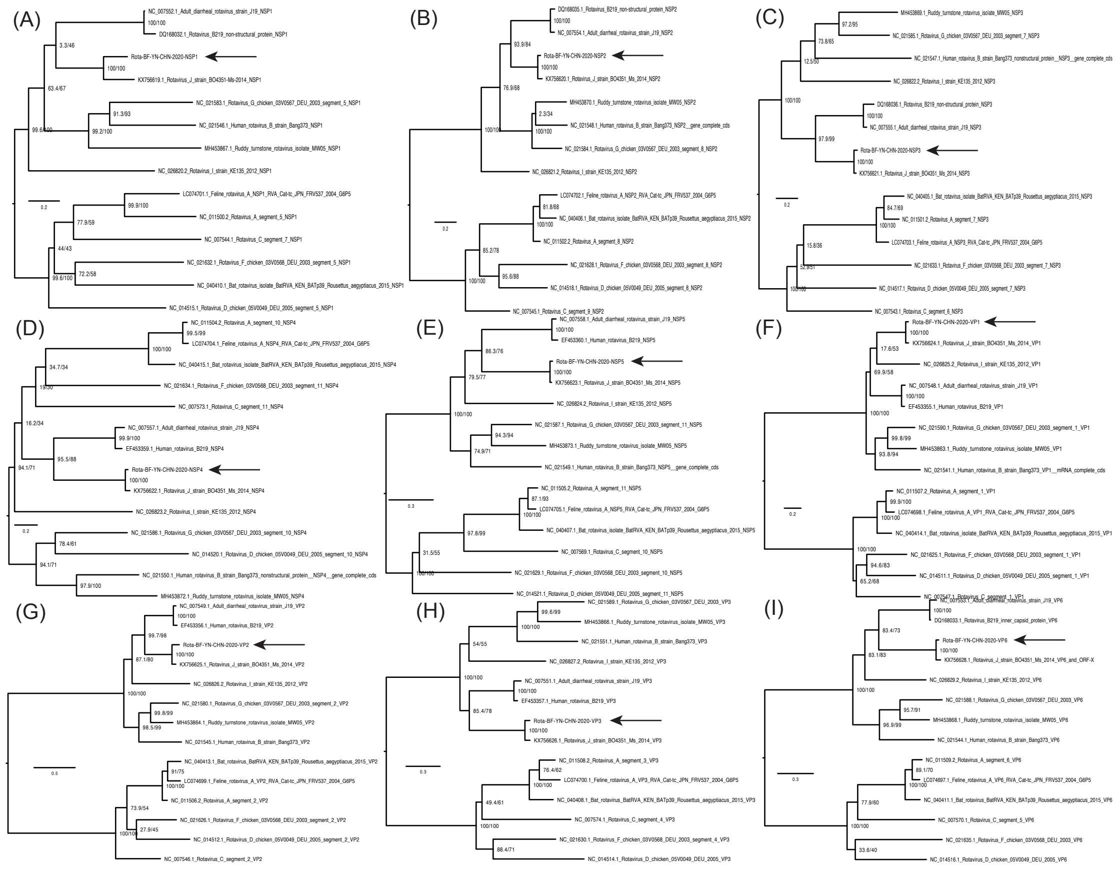

Fig S6. Characterization of the rotavirus genome contigs. (A) The top two hits in the nucleotide (NT) and protein (NR) databases obtained using BLASTN and BLASTP, based on the VP6 segment of a newly identified rotavirus. (B) Assignment and characteristics of the genome segments of a newly identified bat rotavirus. Maximum likelihood trees of different rotavirus species and bat rotaviruses based on the amino acid sequences of VP6 (C) and VP1 (D) segments. The black arrows represent the strains identified in this study. Each colour module represents a different rotavirus species. The scale bars show the substitutions per site per year. The values at each node indicate the bootstrap and SH-like approximate likelihood ratio test (SH-aLRT), with 1000 bootstrap replicates.

(A)

Database	Subject hit	Query cover	Identity
nt	KX756628.1	99%	94.18%
	KX362552.1	27%	66.67%%
nr	KX756628.1	100%	99.75%
	KX362541.1	100%	49.24%

(B)

Segment	Position of start codons	Position of stop codons	Conserved sequences at segment termini	
			5' end	3'end
NSP1	44	1246	GAAA	AATACACCC
NSP2	51	950	GAAA	AATACACCC
NSP3	35	1030	GAAA	AATACACCC
NSP4	21	653	GAAA	AATATACCC
NSP5	52	549	GAAA	AATACACCC
VP1	1	3507	GAAA	AATATACCC
VP2	20	2980	GAAA	AATACACCC
VP3	3	2147	GAAA	AATACACCC
VP6	28	1215	GAAA	AATATACCC

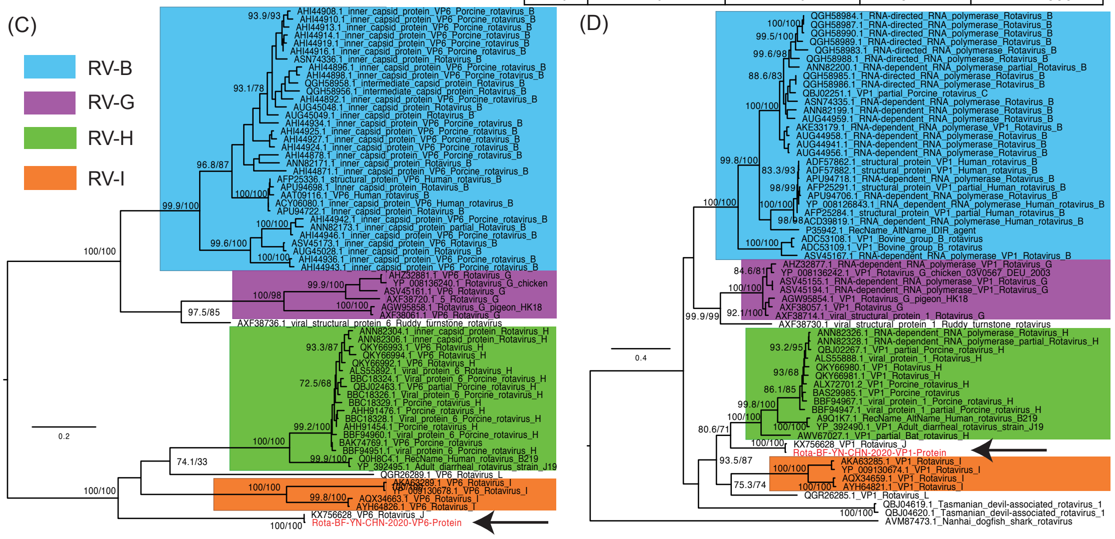

Fig S7. Characterization of the calicivirus genome contigs. (A) Genomic organization of calicivirus and annotated conserved domains. (B) Maximum likelihood tree of the complete VP1 amino acid sequences, including the reference genomes of all *Caliciviridae* and the two newly identified strains in this study. The scale bars show the substitutions per site per year, and the values at each node indicate the bootstrap and SH-like approximate likelihood ratio tests (SH-aLRT), with 1000 bootstrap replicates. The black arrows represent the newly identified strains.

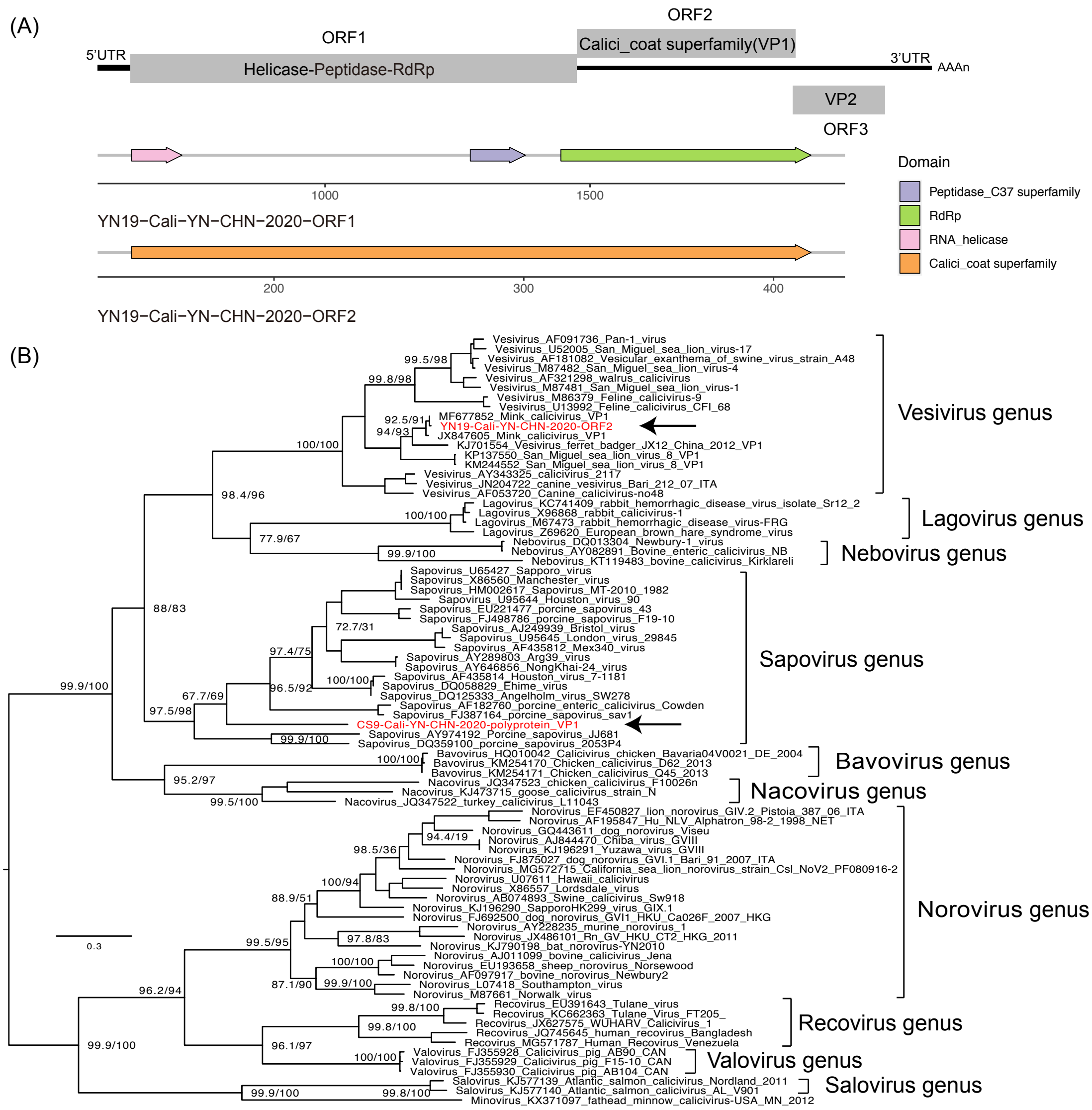

Fig S8. (A) Genomic organization of caliciviruses and annotated conserved domains. (B) Maximum likelihood tree of the full-length polyprotein sequences of neighbouring genomes and the newly identified strain. The scale bars show the substitutions per site per year, and the values at each node indicate the bootstrap and SH-like approximate likelihood ratio tests (SH-aLRT), with 1000 bootstrap replicates. The black arrows represent the newly identified strains.

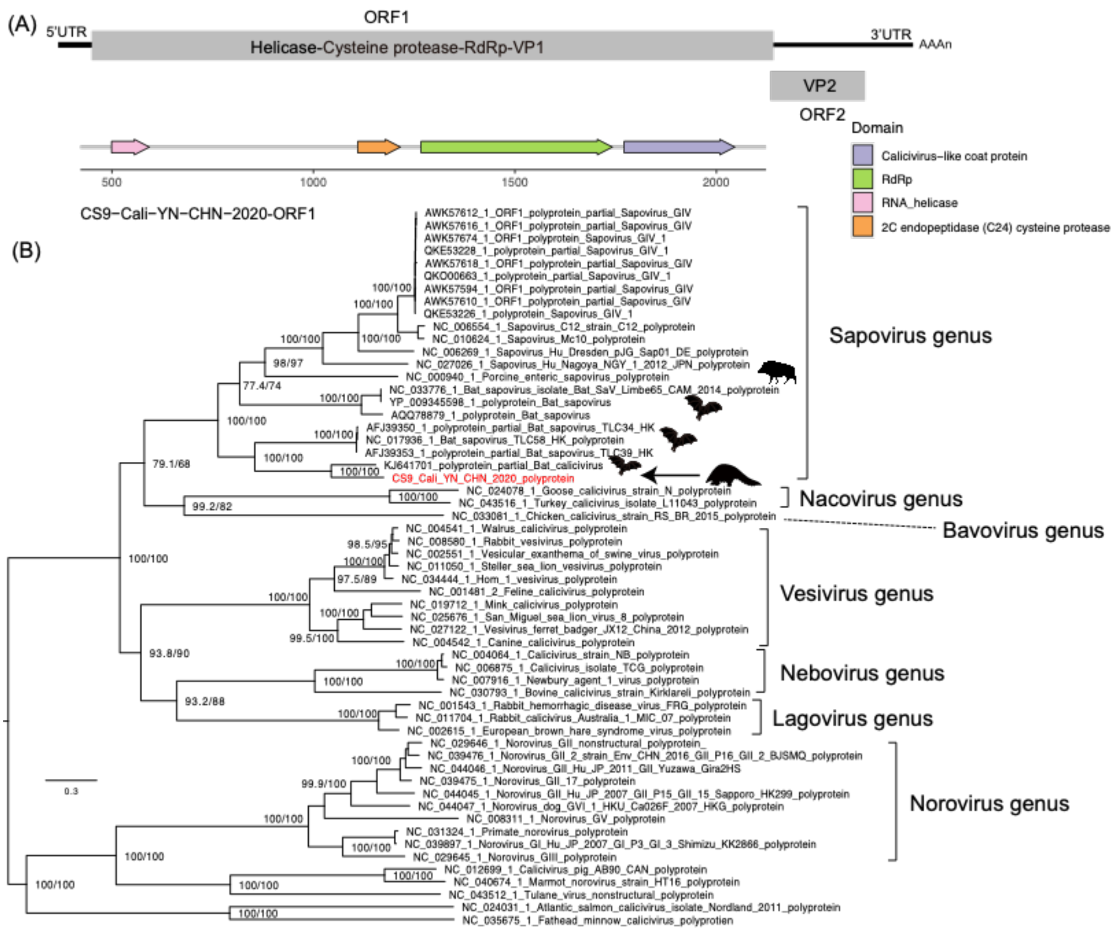

Fig S9. (A) Genomic organization of kobuvirus-related viruses and annotated conserved domains. (B) The top two hits in the nucleotide (NT) and protein (NR) databases using BLASTN and BLASTP. (C) Maximum likelihood tree of the complete amino acid sequences of the reference genomes of known species of kobuvirus and neighbouring strains. The scale bars show the substitutions per site per year, and the values at each node indicate the bootstrap and SH-like approximate likelihood ratio tests (SH-aLRT), with 1000 bootstrap replicates. The black arrows represent the newly identified strains.

(A)

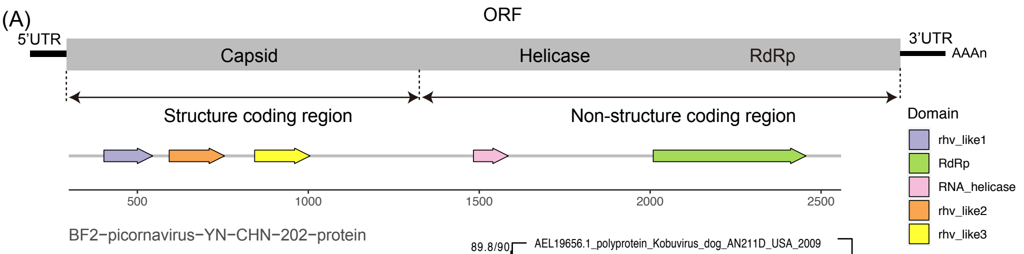

(B)

Database	Subject hit	Query cover	Identity
nt	MF352427.1	100%	96.98%
	KJ641691.1	88%	96.74%%
nr	MF352427.1	97%	99.71%
	KJ641691.1	94%	99.36%

(C)

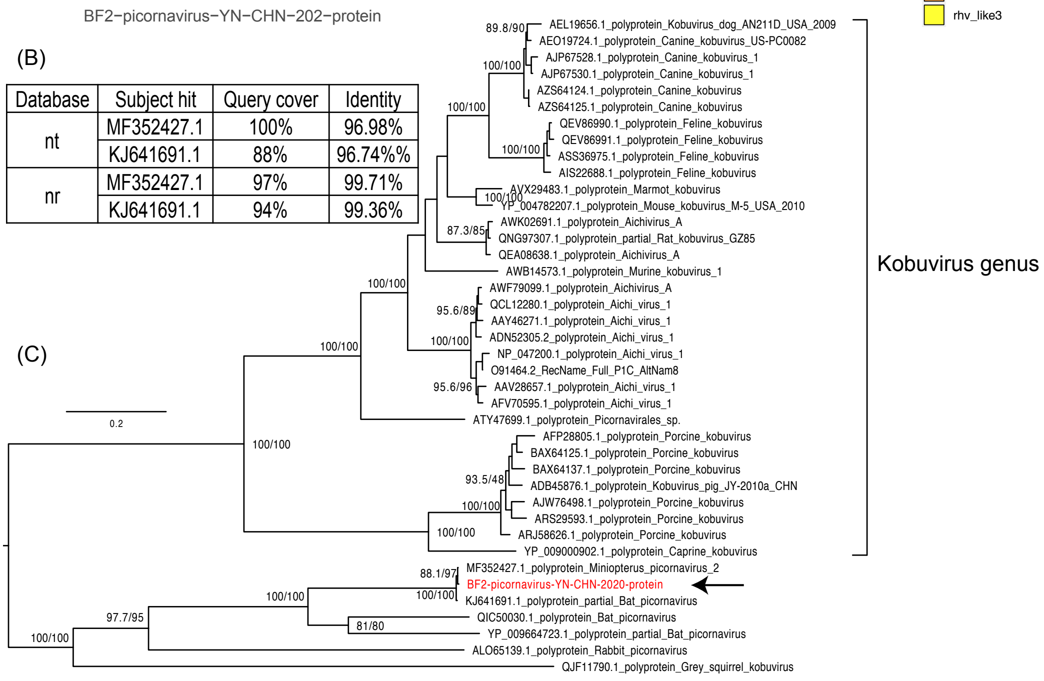

Fig S10. (A) Genomic organization of the newly identified kobuvirus in this study and the mapped region. (B) The top two hits in the nucleotide (NT) and protein (NR) database using BLASTN and BLASTP. (C) Maximum likelihood tree of the complete P1 coding region sequences, including the reference genomes of known species of kobuvirus and the newly identified strain. The scale bars show the substitutions per site per year, and the values at each node indicate the bootstrap and SH-like approximate likelihood ratio tests (SH-aLRT), with 1000 bootstrap replicates. The black arrows represent the newly identified strains.

(A)

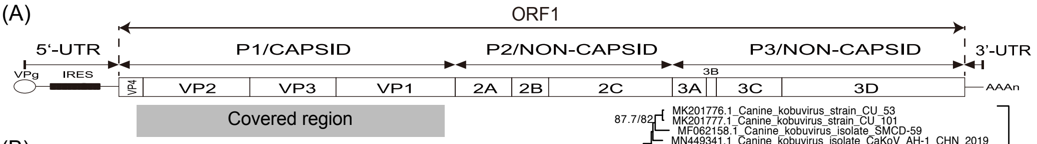

(B)

Database	Subject hit	Query cover	Identity
nt	MF947438.1	99%	86.95%
	MF947440.1	99%	86.90%
nr	MF947438.1	99%	96.93%
	MF947439.1	99%	96.93%

(C)

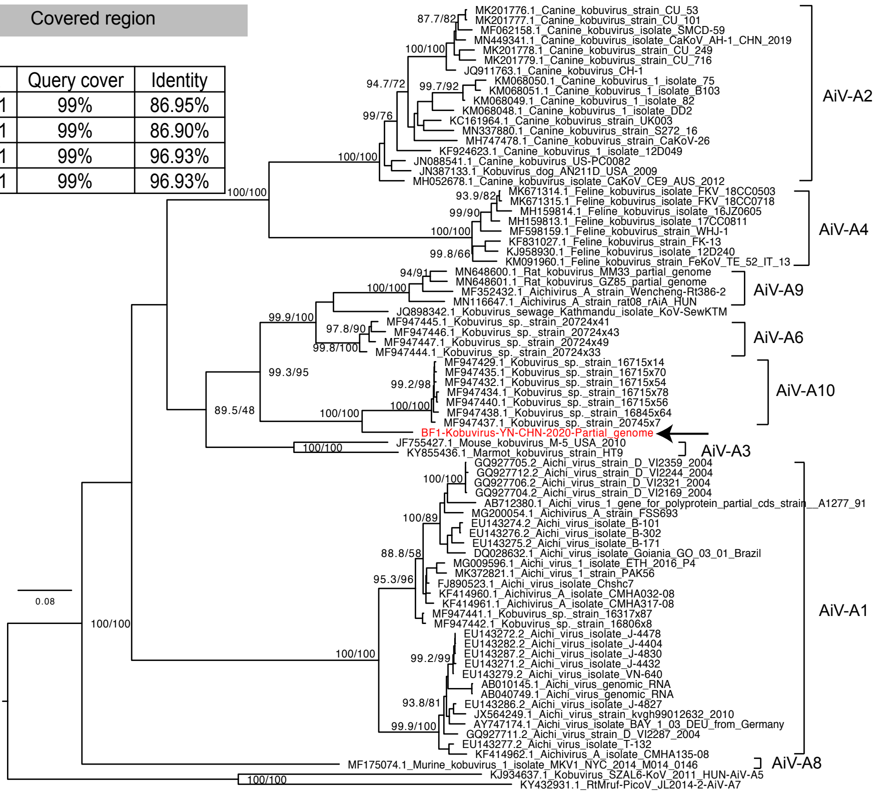

Fig S11. (A) Genomic organization of the newly identified EV-A122 strains in this study. Maximum likelihood tree of the complete P1 (B) and P3 (C) coding region sequences, including the reference genomes of known serotypes of enterovirus A and the newly identified strains. The scale bars show the substitutions per site per year, and the values at each node indicate the bootstrap and SH-like approximate likelihood ratio tests (SH-aLRT), with 1000 bootstrap replicates. The black arrows represent the newly identified strains. (D) Similarity plot of the circulating recombination donors and newly identified strains. The genomic map (upper) and recombination events predicted for the strains in this study, which is shown as a black block. Genetic components identified by RDP4, which were involved in the recombination events, are shown as different colour blocks. Likely breakpoint positions are shown above the genome.

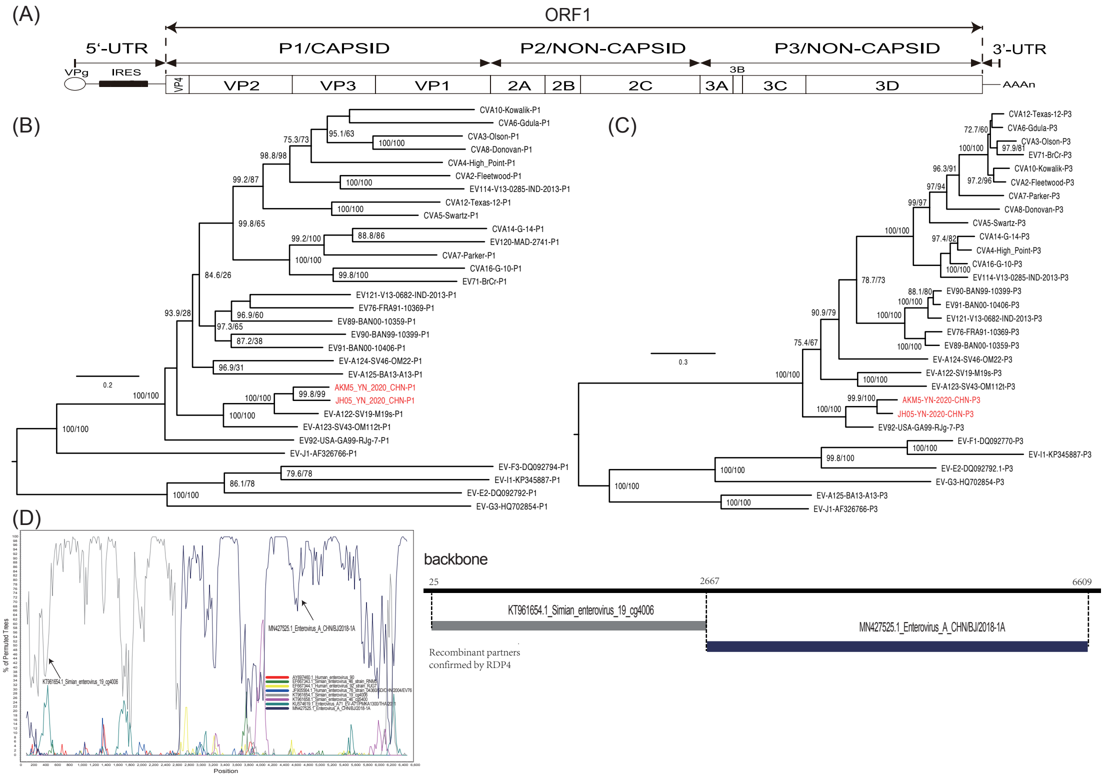

Fig S12. Characterization of the posa-like virus genome contigs. (A) Genomic organization of posa-like viruses and annotated conserved domains. (B) Maximum likelihood tree of the complete amino acid sequences, including the reference genomes of known species of posa-like virus and neighbouring strains. The scale bars show the substitutions per site per year, and the values at each node indicate the bootstrap and SH-like approximate likelihood ratio tests (SH-aLRT), with 1000 bootstrap replicates. The black arrows represent the newly identified strains.

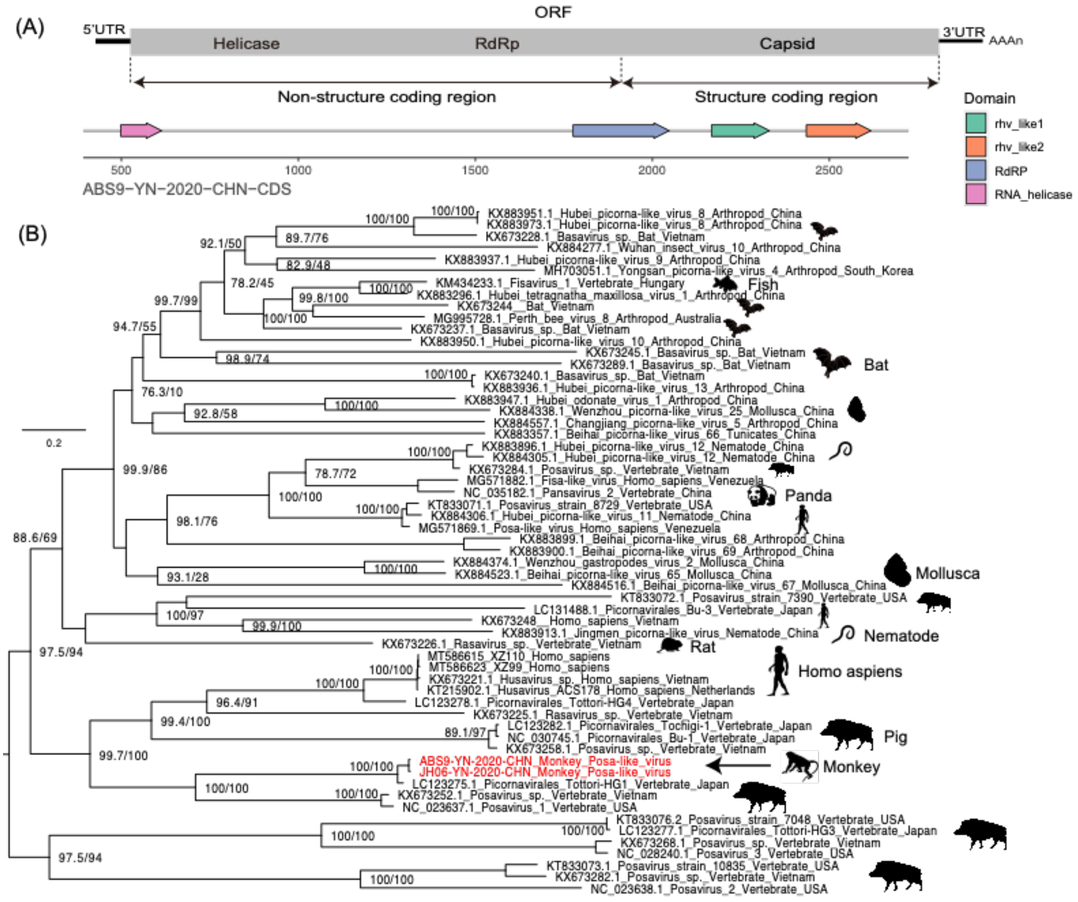

Supplement: Supplementary Figure 1 — Genomic organization and similarity plots of two representative coronavirus strains identified in this study, strain ATG20-YN-CHN-2020 (A) and strain ATG32-YN-CHN-2020. The phylogenetic neighbors were selected for the similarity plot. The black line indicates 90% similarity. [file Data_Sheet_1.PDF]
